# Supplementary material for: Inhibition of osteoblastic Smurf1 promotes bone formation in mouse models of distinctive age-related osteoporosis
Source: Nat Commun. 2018 Aug 24;9:3428. doi: 10.1038/s41467-018-05974-z (PMC6109183; doi:10.1038/s41467-018-05974-z)
Supplement: Supplementary file 1 — Supplementary Information [file 41467_2018_5974_MOESM1_ESM.pdf]

Supplementary Information

Inhibition of osteoblastic Smurf1 promotes bone formation in mouse models of distinctive age-related osteoporosis

Liang *et. al.*

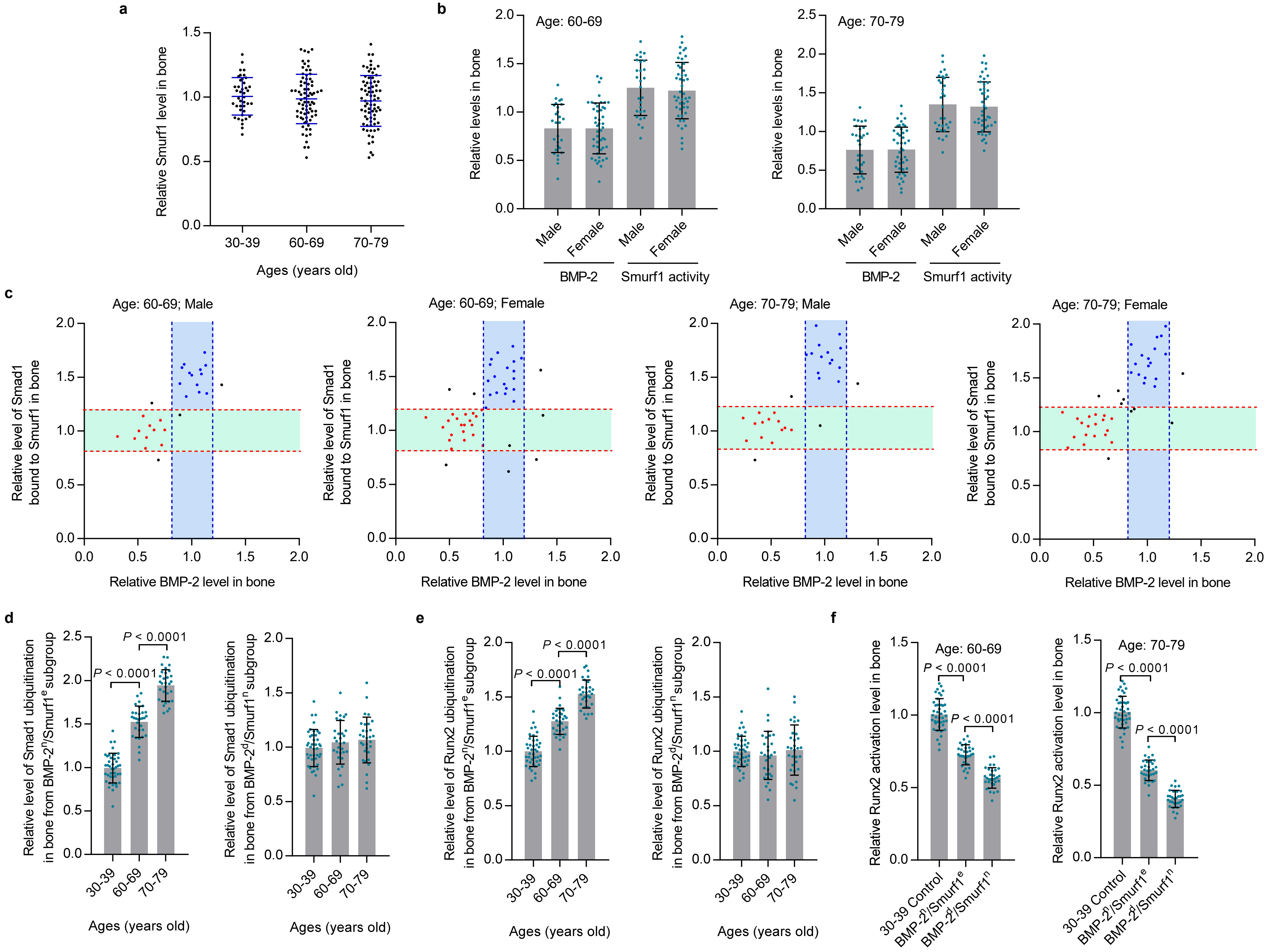
a
Relative Smurf1 level in bone
Ages (years old)
30-39
60-69
70-79
b
Age: 60-69
Age: 70-79
Relative levels in bone
Male
Female
BMP-2
Smurf1 activity
c
Age: 60-69; Male
Age: 60-69; Female
Age: 70-79; Male
Age: 70-79; Female
Relative level of Smad1 bound to Smurf1 in bone
Relative BMP-2 level in bone
d
Relative level of Smad1 ubiquitination in bone from BMP-2n/Smurf1e subgroup
Relative level of Smad1 ubiquitination in bone from BMP-2d/Smurf1n subgroup
P < 0.0001
Ages (years old)
e
Relative level of Runx2 ubiquitination in bone from BMP-2n/Smurf1e subgroup
Relative level of Runx2 ubiquitination in bone from BMP-2d/Smurf1n subgroup
f
Relative Runx2 activation level in bone
30-39 Control
BMP-2n/Smurf1e
BMP-2d/Smurf1n

Supplementary Figure 1. Smurf1 activity and osteogenic potential in BMP-2^{n}/Smurf1^{e} and BMP-2^{d}/Smurf1^{n} subgroups of aged osteoporotic VCF patients. **(a)** Relative level of intraosseous Smurf1 in osteoporotic VCF patients aged 60-69 (n = 75) or 70-79 years old (n = 75). **(b)** Relative levels of intraosseous BMP-2 and Smurf1 activity (Smad1 bound to Smurf1) in aged male or female osteoporotic VCF patients (left, 60-69 age group; right, 70-79 age group; n = 27 for male patients and n = 48 for female patients in 60-69 age group; n = 31 for male patients and n = 44 for female patient in 70-79 age group). **(c)** Classification of male or female osteoporotic VCF patients into subgroups based on distinct intraosseous BMP-2 levels and Smurf1 activity (Smad1 bound to Smurf1) (left, male patients in 60-69 age group; middle left, female patients in 60-69 age group; middle right, male patients in 70-79 age group; right, female patients in 70-79 age group). Relative levels of intraosseous BMP-2 (mean ± s.d., indicated by the two blue dashed lines) and Smad1 bound to Smurf1 (mean ± s.d., indicated by the two red dashed lines) in adult traumatic VCF patients (30-39 years old) served as cutoff parameters. **(d)** Relative level of intraosseous Smad1 ubiquitination in BMP-2^{n}/Smurf1^{e} subgroup (left; n = 32 for 60-69 years old; n = 31 for 70-79 years old) and BMP-2^{d}/Smurf1^{n} subgroup (right; n = 31 for 60-69 years old; n = 31 for 70-79 years old). **(e)** Relative level of intraosseous Runx2 ubiquitination in the above two subgroups. **(f)** Relative level of intraosseous Runx2 activation in the above two subgroups of aged osteoporotic VCF patients (left, 60-69 years old; right, 70-79 years old). The levels of Smurf1 and BMP-2, Smad1 bound to Smurf1, Runx2 activation and ubiquitination of Smad1 and Runx2 were normalized to the mean values of adult traumatic VCF patients (30-39 years old) (n = 41). Data are mean ± s.d. followed by one-way ANOVA with a *post-hoc* test or Student's *t* test.

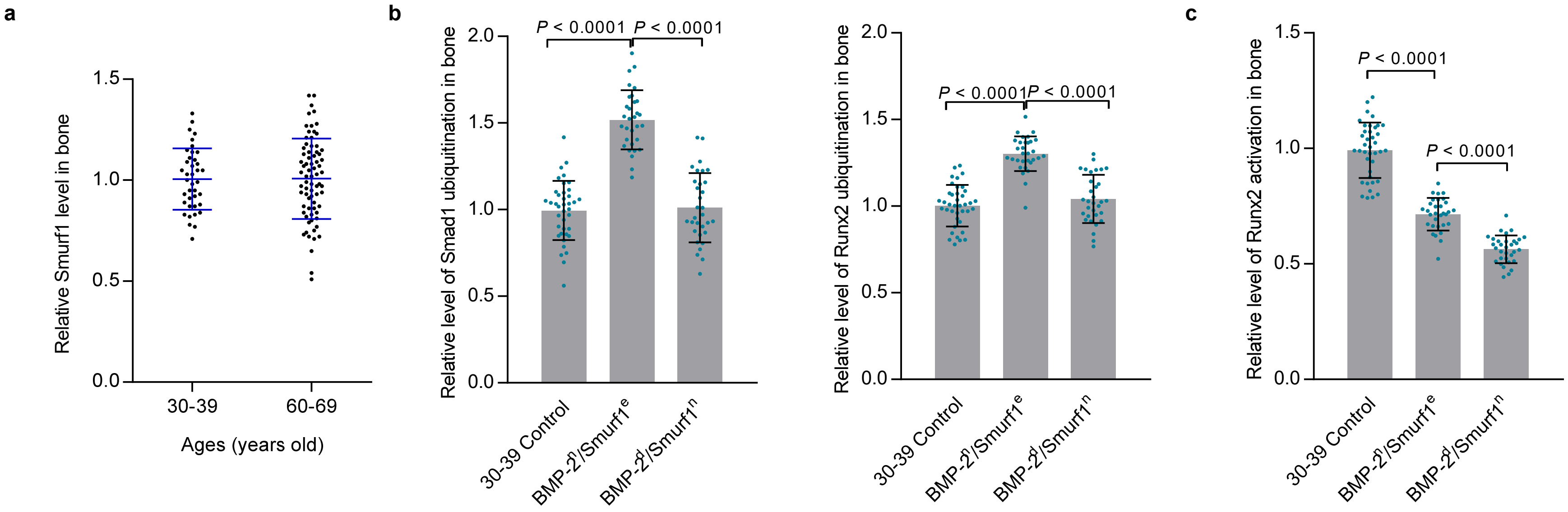

Supplementary Figure 2. Smurf1 activity and osteogenic potential in BMP-2^{n}/Smurf1^{e} and BMP-2^{d}/Smurf1^{n} subgroups of aged osteoporotic LSS patients. (a) Relative level of intraosseous Smurf1 in osteoporotic LSS patients aged 60-69 years old (n = 74). **(b)** Relative levels of intraosseous Smad1 and Runx2 ubiquitination in BMP-2^{n}/Smurf1^{e} subgroup (n = 31) and BMP-2^{d}/Smurf1^{n} subgroup (n = 32). **(c)** Relative level of intraosseous Runx2 activation in the above two subgroups. The levels of Smurf1, Runx2 activation and ubiquitination of Smad1 and Runx2 were normalized to the mean values of adult LDH patients (30-39 years old) (n = 39). Data are mean ± s.d. followed by one-way ANOVA with a *post-hoc* test or Student's *t* test.

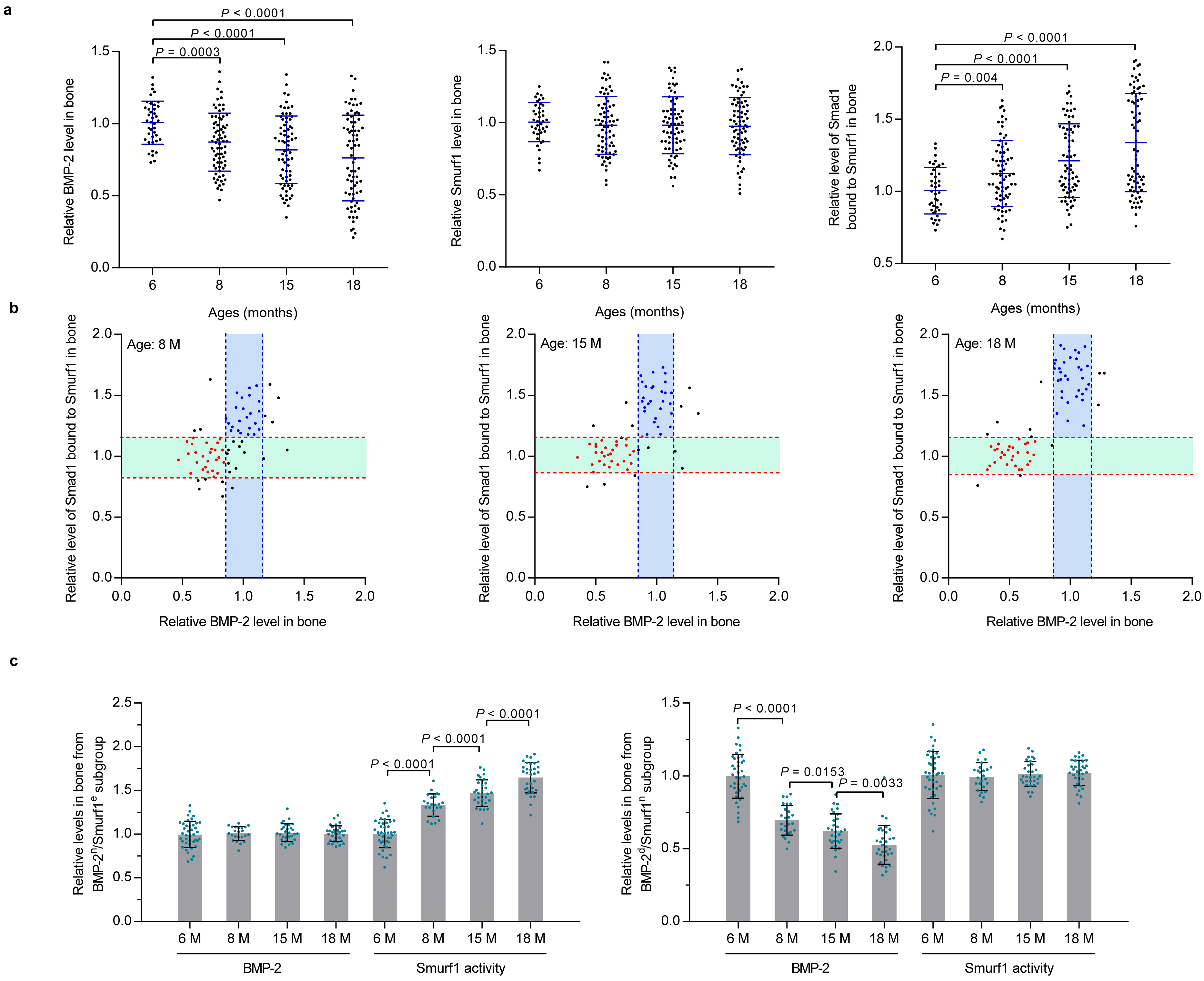

a
P < 0.0001
P < 0.0001
P = 0.0003
Relative BMP-2 level in bone
Relative Smurf1 level in bone
P = 0.004
Relative level of Smad1 bound to Smurf1 in bone
Ages (months)
b
Age: 8 M
Age: 15 M
Age: 18 M
Relative level of Smad1 bound to Smurf1 in bone
Relative BMP-2 level in bone
c
Relative levels in bone from BMP-2^n/Smurf1^e subgroup
Relative levels in bone from BMP-2^d/Smurf1^n subgroup
P = 0.0153
P = 0.0033
6 M
8 M
15 M
18 M
BMP-2
Smurf1 activity

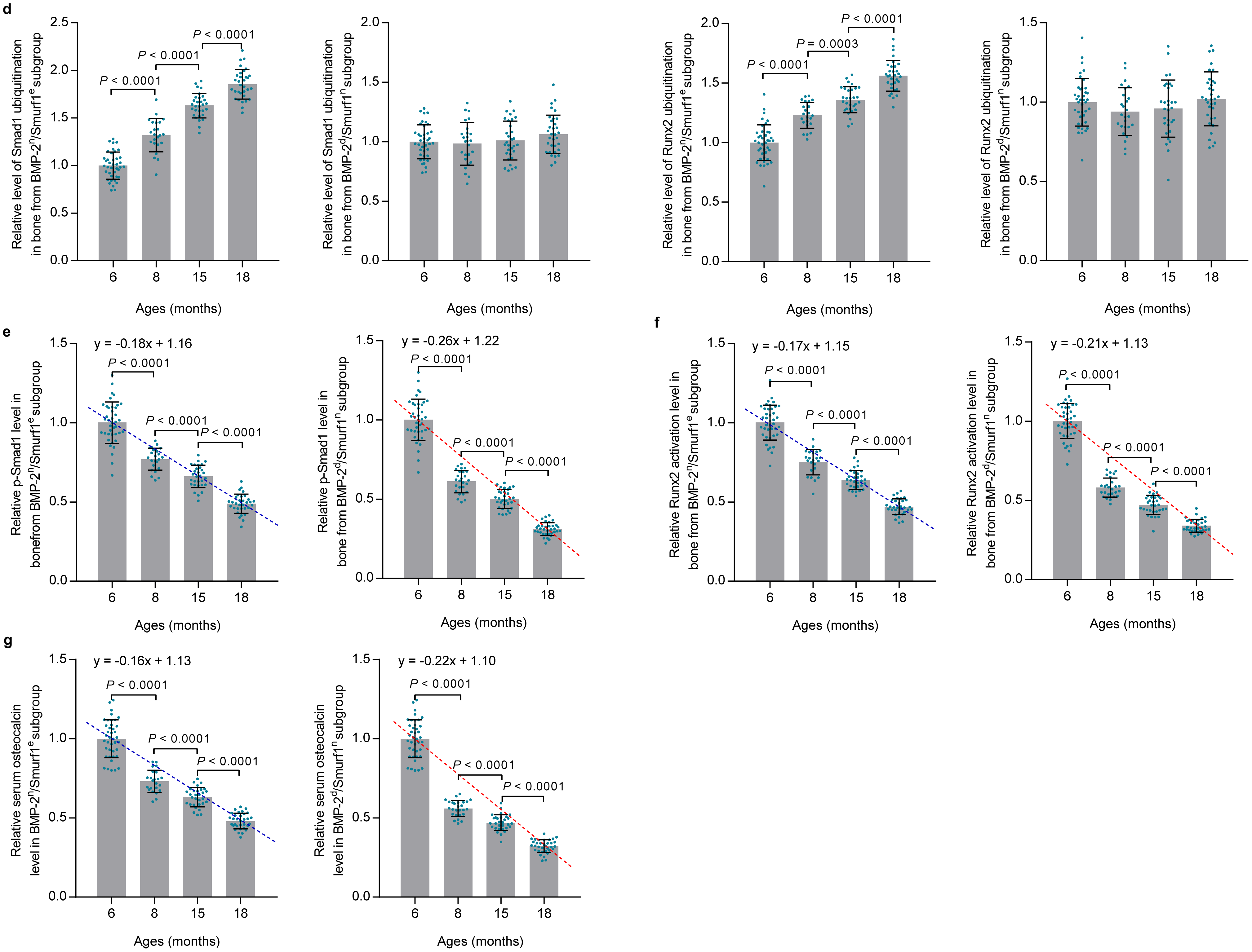
d
Relative level of Smad1 ubiquitination in bone from BMP-2^{n}/Smurf1^{e} subgroup
P < 0.0001
P < 0.0001
P < 0.0001
Relative level of Smad1 ubiquitination in bone from BMP-2^{d}/Smurf1^{n} subgroup
Relative level of Runx2 ubiquitination in bone from BMP-2^{n}/Smurf1^{e} subgroup
P < 0.0001
P = 0.0003
P < 0.0001
Relative level of Runx2 ubiquitination in bone from BMP-2^{d}/Smurf1^{n} subgroup
Ages (months)
6
8
15
18
e
y = -0.18x + 1.16
Relative p-Smad1 level in bonefrom BMP-2^{n}/Smurf1^{e} subgroup
y = -0.26x + 1.22
Relative p-Smad1 level in bone from BMP-2^{d}/Smurf1^{n} subgroup
f
y = -0.17x + 1.15
Relative Runx2 activation level in bone from BMP-2^{n}/Smurf1^{e} subgroup
y = -0.21x + 1.13
Relative Runx2 activation level in bone from BMP-2^{d}/Smurf1^{n} subgroup
g
y = -0.16x + 1.13
Relative serum osteocalcin level in BMP-2^{n}/Smurf1^{e} subgroup
y = -0.22x + 1.10
Relative serum osteocalcin level in BMP-2^{d}/Smurf1^{n} subgroup
P < 0.0001

Supplementary Figure 3. BMP signaling, Smurf1 activity and osteogenic potential in subgroups of aged osteoporotic mice. (a) Levels of intraosseous BMP-2 (left), Smurf1 (middle) and Smurf1 activity (Smad1 bound to Smurf1, right) in osteoporotic mice (*n* = 73 for 8-month-old; *n* = 72 for 15-month-old; n = 75 for 18-month-old). 6-month-old female mice were OVX and left untreated for 2 months (8-month-old), 9 months (15-month-old) or 12 months (18-month-old) to induce age-related osteoporosis. **(b)** Classification of osteoporotic mice into subgroups based on distinct intraosseous levels of BMP-2 and Smurf1 activity (Smad1 bound to Smurf1) (left, 8-month-old; middle, 15-month-old; right, 18-month-old). The cluster of blue dots, BMP-2^n/Smurf1^e subgroup; the cluster of red dots, BMP-2^d/Smurf1^n subgroup. Relative levels of intraosseous BMP-2 (mean ± s.d., indicated by the two blue dashed lines) and Smad1 bound to Smurf1 (mean ± s.d., indicated by the two red dashed lines) in healthy adult 6-month-old mice served as cutoff parameters. **(c)** Relative levels of intraosseous BMP-2 and Smurf1 activity (Smad1 bound to Smurf1) in BMP-2^n/Smurf1^e subgroup (left; n = 23 for 8-month-old; *n* = 30 for 15-month-old; *n* = 32 for 18-month-old) and BMP-2^d/Smurf1^n subgroup (right; *n* = 26 for 8-month-old; *n* = 31 for 15-month-old; *n* = 34 for 18-month-old), respectively. **(d)** Relative levels of intraosseous Smad1 and Runx2 ubiquitination in the above two subgroups. **(e)** Relative level of intraosseous p-Smad1 in the above two subgroups. Blue dashed line: age-related change in BMP-2^n/Smurf1^e subgroup; red dashed line: age-related change in BMP-2^d/Smurf1^n subgroup. **(f)** Relative level of intraosseous Runx2 activation in the above two subgroups. Blue dashed line: age-related change in BMP-2^n/Smurf1^e subgroup; red dashed line: age-related change in BMP-2^d/Smurf1^n subgroup. **(g)** Relative level of serum osteocalcin in the above two subgroups. Blue dashed line, age-related change in BMP-2^n/Smurf1^e subgroup; red dashed line, age-related change in BMP-2^d/Smurf1^n subgroup. The levels of BMP-2, p-Smad1 and osteocalcin, Runx2 activation, Smad1 bound to Smurf1 and ubiquitination of Smad1 and Runx2 were normalized to the mean values of healthy adult 6-month-old mice (*n* = 40). Data are mean ± s.d. followed by one-way ANOVA with a *post-hoc* test.

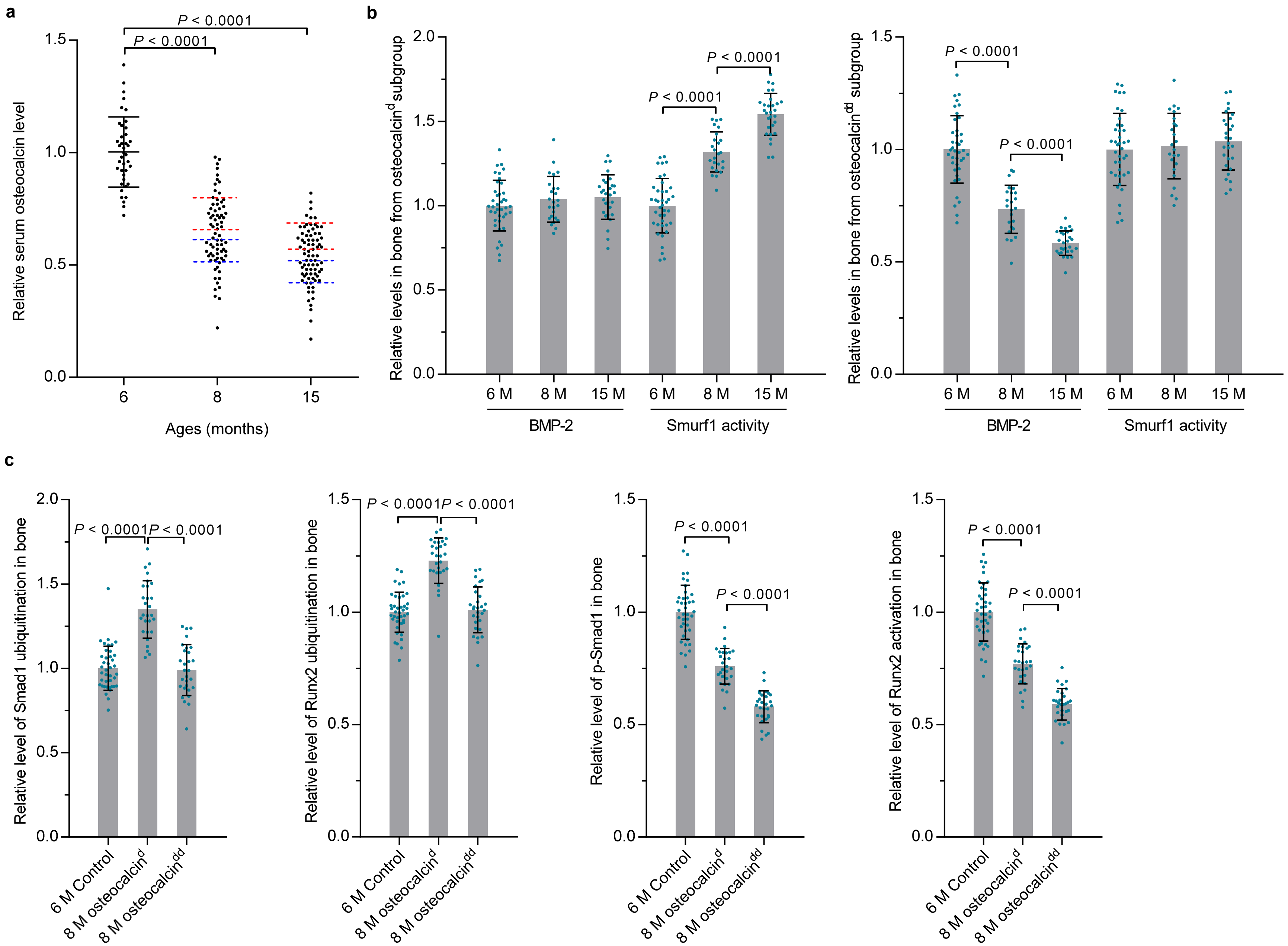
a
P < 0.0001
P < 0.0001
Relative serum osteocalcin level
Ages (months)
b
Relative levels in bone from osteocalcin^d subgroup
P < 0.0001
P < 0.0001
6 M
8 M
15 M
BMP-2
Smurf1 activity
Relative levels in bone from osteocalcin^dd subgroup
P < 0.0001
P < 0.0001
c
Relative level of Smad1 ubiquitination in bone
Relative level of Runx2 ubiquitination in bone
Relative level of p-Smad1 in bone
Relative level of Runx2 activation in bone
P < 0.0001
6 M Control
8 M osteocalcin^d
8 M osteocalcin^dd

d

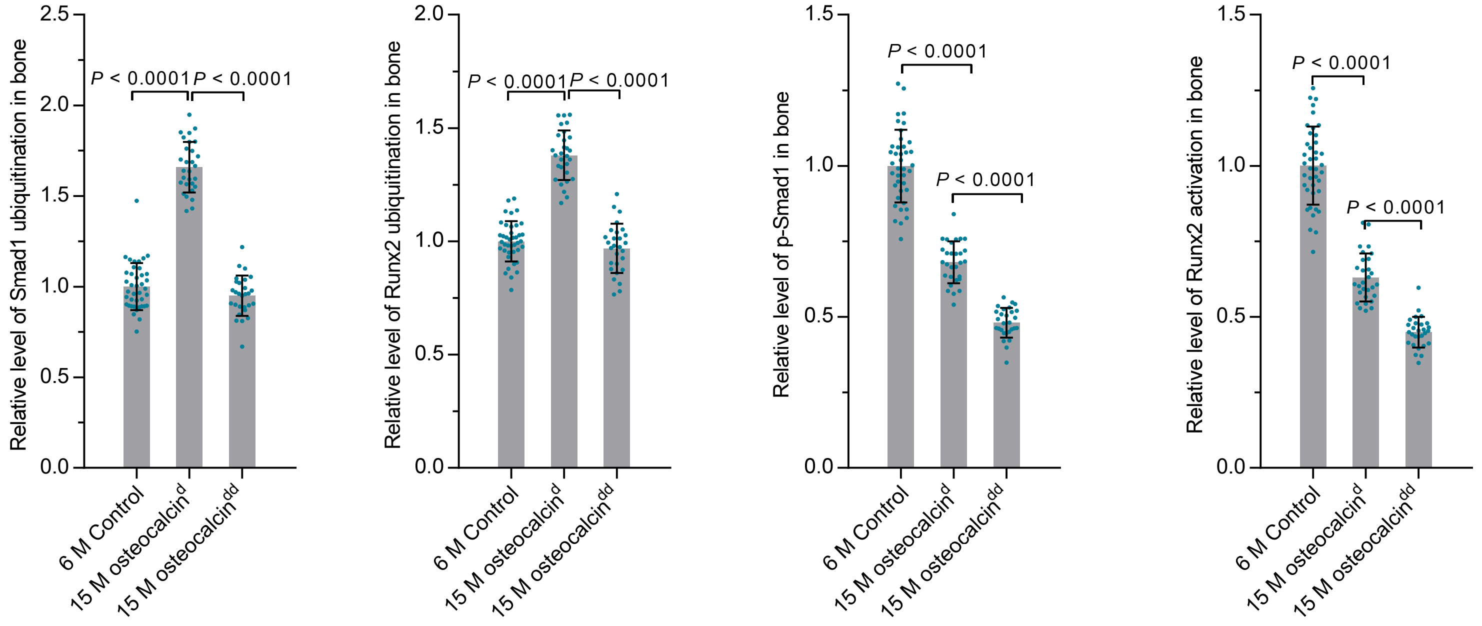

Supplementary Figure 4. Serum osteocalcin as a biomarker in stratifying mice BMP-2^{n}/Smurf1^{e} and BMP-2^{d}/Smurf1^{n} subgroups. (a) Relative level of serum osteocalcin in osteoporotic mice aged 8 months old (n = 73) and 15 months old (n = 78). Osteocalcin^{d} subgroup (the cluster of dots between the two red dashed lines), a major subgroup with a comparable serum osteocalcin level to that in BMP-2^{n}/Smurf1^{e} subgroup (0.73 ± 0.07 for 8-month-old osteoporotic mice; 0.63 ± 0.06 for 15-month-old osteoporotic mice); osteocalcin^{dd} subgroup (the cluster of dots between the two blue dashed lines), another major subgroup with an equivalent serum osteocalcin level to that in BMP-2^{d}/Smurf1^{n} subgroup (0.56 ± 0.05 for 8-month-old osteoporotic mice; 0.47 ± 0.05 for 15-month-old osteoporotic mice). **(b)** Relative levels of BMP-2 and Smurf1 activity (Smad1 bound to Smurf1) in osteocalcin^{d} subgroup (n = 25 for 8-month-old; n = 29 for 15-month-old) and osteocalcin^{dd} subgroup (n = 24 for 8-month-old; n = 28 for 15-month-old). **(c)** Relative levels of Smad1 and Runx2 ubiquitination, p-Smad1 and Runx2 activation in osteocalcin^{d} subgroup (n = 28) and osteocalcin^{dd} subgroup (n = 29) of 8-month-old osteoporotic mice. **(d)** Relative levels of Smad1 and Runx2 ubiquitination, p-Smad1 and Runx2 activation in osteocalcin^{d} subgroup (n = 29) and osteocalcin^{dd} subgroup (n = 28) of 15-month-old osteoporotic mice. 6-month-old female mice were OVX and left untreated for 2 months (8-month-old) or 9 months (15-month-old) to induce age-related osteoporosis. The levels of BMP-2, p-Smad1 and osteocalcin, Runx2 activation, Smad1 bound to Smurf1 and ubiquitination of Smad1 and Runx2 were normalized to the mean values of healthy adult 6-month-old mice (n = 40). Data are mean ± s.d. followed by one-way ANOVA with a *post-hoc* test.

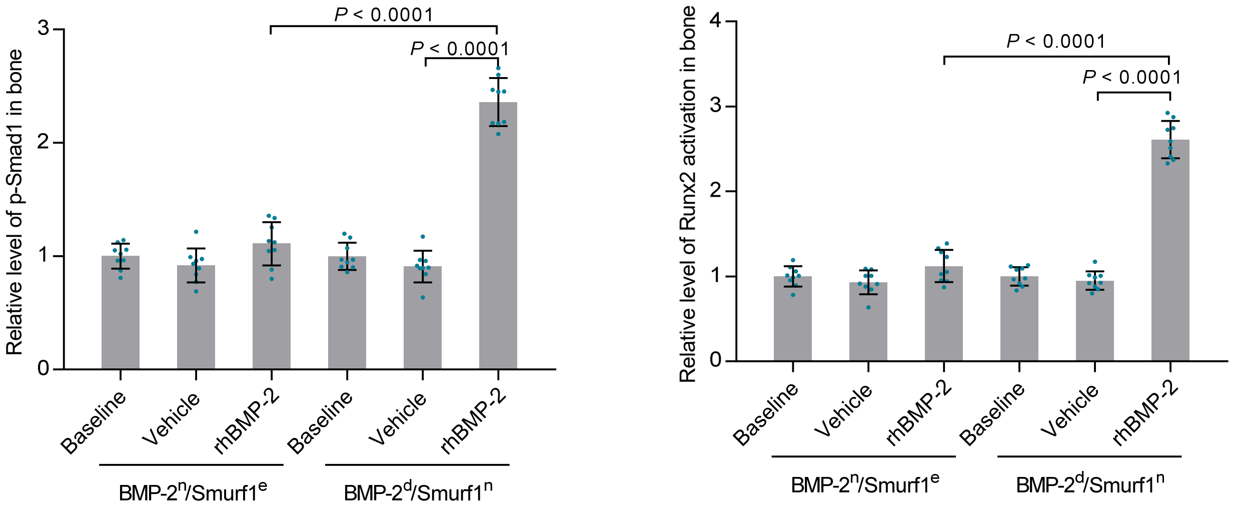

Supplementary Figure 5. Local response to rhBMP-2 in osteoblasts from mice BMP-2^{n}/Smurf1^{e} and BMP-2^{d}/Smurf1^{n} subgroups. Relative levels of p-Smad1 (left) and Runx2 activation (right) in osteoblasts from L4-L6 vertebrae of BMP-2^{n}/Smurf1^{e} and BMP-2^{d}/Smurf1^{n} subgroups of 15-month-old osteoporotic mice (OVX at 6 months old) with local administration of rhBMP-2 or vehicle (PBS). The levels of p-Smad1 and Runx2 activation in osteoblasts from L4-L6 vertebrae of BMP-2^{n}/Smurf1^{e} and BMP-2^{d}/Smurf1^{n} subgroups were normalized to their corresponding mean value of vehicle baseline. n = 9 per group. Data are mean ± s.d. followed by one-way ANOVA with a *post-hoc* test.

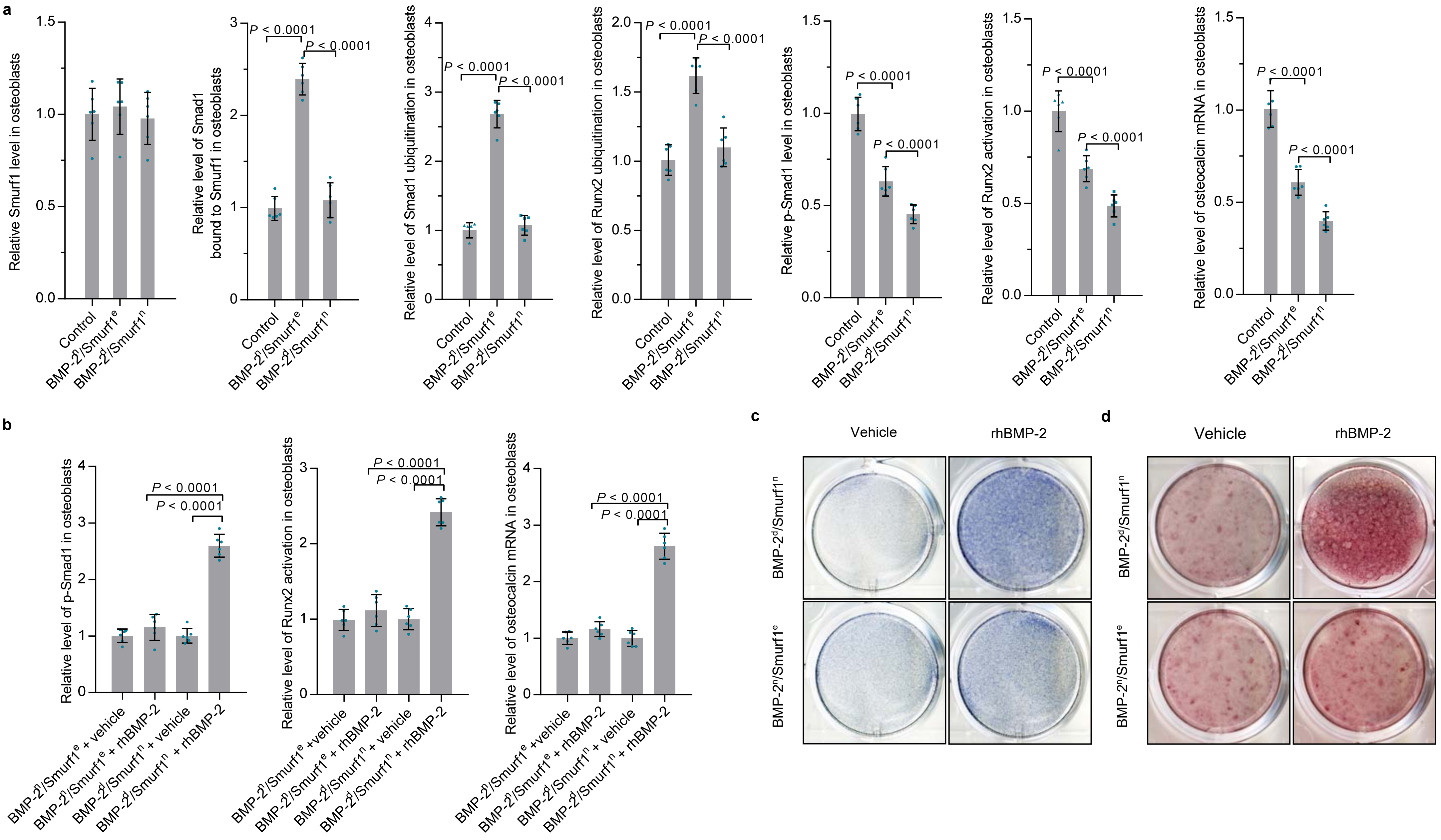

a
Relative Smurf1 level in osteoblasts
Relative level of Smad1 bound to Smurf1 in osteoblasts
Relative level of Smad1 ubiquitination in osteoblasts
Relative level of Runx2 ubiquitination in osteoblasts
Relative p-Smad1 level in osteoblasts
Relative level of Runx2 activation in osteoblasts
Relative level of osteocalcin mRNA in osteoblasts
P < 0.0001
Control
BMP-2d/Smurf1e
BMP-2d/Smurf1n
b
Relative level of p-Smad1 in osteoblasts
Relative level of Runx2 activation in osteoblasts
Relative level of osteocalcin mRNA in osteoblasts
P < 0.0001
BMP-2n/Smurf1e + vehicle
BMP-2n/Smurf1e + rhBMP-2
BMP-2d/Smurf1n + vehicle
BMP-2d/Smurf1n + rhBMP-2
c
Vehicle
rhBMP-2
BMP-2d/Smurf1n
BMP-2n/Smurf1e
d
Vehicle
rhBMP-2
BMP-2d/Smurf1n
BMP-2n/Smurf1e

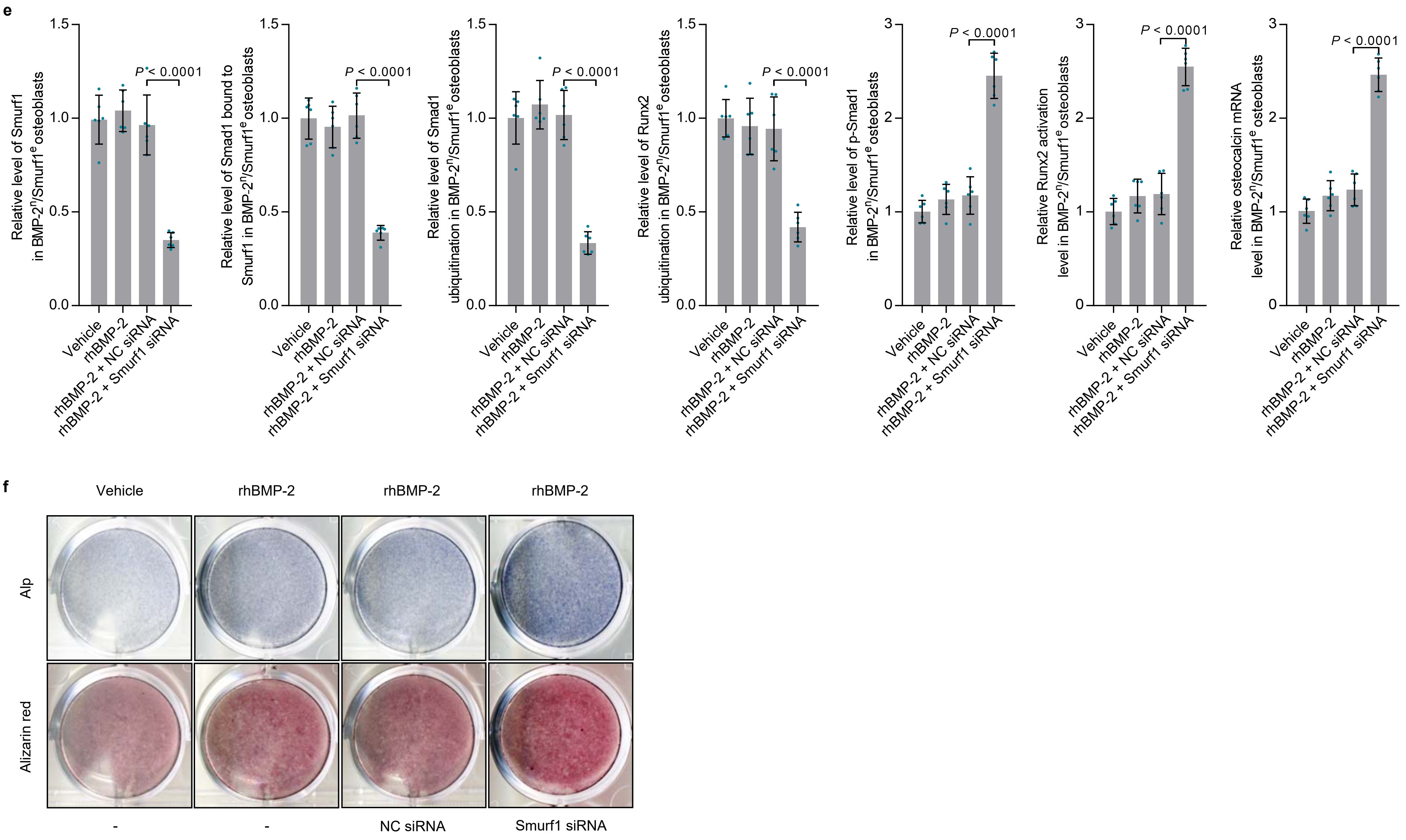

e
Relative level of Smurf1 in BMP-2ⁿ/Smurf1ᵉ osteoblasts
Relative level of Smad1 bound to Smurf1 in BMP-2ⁿ/Smurf1ᵉ osteoblasts
Relative level of Smad1 ubiquitination in BMP-2ⁿ/Smurf1ᵉ osteoblasts
Relative level of Runx2 ubiquitination in BMP-2ⁿ/Smurf1ᵉ osteoblasts
Relative level of p-Smad1 in BMP-2ⁿ/Smurf1ᵉ osteoblasts
Relative Runx2 activation level in BMP-2ⁿ/Smurf1ᵉ osteoblasts
Relative osteocalcin mRNA level in BMP-2ⁿ/Smurf1ᵉ osteoblasts
P < 0.0001
0.0
0.5
1.0
1.5
0
1
2
3
Vehicle
rhBMP-2
rhBMP-2 + NC siRNA
rhBMP-2 + Smurf1 siRNA
f
Vehicle
rhBMP-2
Alp
Alizarin red
-
NC siRNA
Smurf1 siRNA

Supplementary Figure 6. Effects of Smurf1 knockdown on BMP signaling in osteoblasts from mice BMP-2^{n}/Smurf1^{e} subgroup. (a) Relative levels of Smurf1 expression, Smurf1 activity (Smad1 bound to Smurf1, ubiquitination of Smad1 and Runx2), p-Smad1, Runx2 activation and osteocalcin mRNA in osteoblasts from BMP-2^{n}/Smurf1^{e} and BMP-2^{d}/Smurf1^{n} subgroups of 15-month-old osteoporotic mice (OVX at 6 months old). The levels of Smurf1 and p-Smad1, Runx2 activation, Smad1 bound to Smurf1, ubiquitination of Smad1 and Runx2 and osteocalcin mRNA were normalized to the mean values of osteoblasts from healthy adult 6-month-old mice. **(b)** Relative levels of p-Smad1, Runx2 activation and osteocalcin mRNA in the above two subgroups of osteoblasts treated with rhBMP-2 or vehicle (PBS). The levels of p-Smad1 and osteocalcin mRNA in BMP-2^{n}/Smurf1^{e} and BMP-2^{d}/Smurf1^{n} subgroups were normalized to their corresponding mean values of osteoblasts treated with vehicle (PBS). **(c)** Alp staining showing Alp activity in the two subgroups of osteoblasts treated with 100 ng ml^{-1} rhBMP-2 or vehicle (PBS). **(d)** Alizarin red staining showing mineralized nodule formation. **(e)** Effects of Smurf1 knockdown on relative levels of Smurf1 activity (Smad1 bound to Smurf1, ubiquitination of Smad1 and Runx2), p-Smad1, Runx2 activation and osteocalcin mRNA in osteoblasts (from BMP-2^{n}/Smurf1^{e} subgroup) treated with 100 ng ml^{-1} rhBMP-2. The levels of Smurf1, Smad1 bound to Smurf1, ubiquitination of Smad1 and Runx2, p-Smad1, Runx2 activation and osteocalcin mRNA were normalized to the mean values of osteoblasts treated with vehicle (PBS). **(f)** Alp (upper) and alizarin red (lower) staining showing effects of Smurf1 knockdown on Alp activity and mineralized nodule formation in osteoblasts (from BMP-2^{n}/Smurf1^{e} subgroup) treated with 100 ng ml^{-1} rhBMP-2. NC siRNA: non-sense siRNA. n = 6 per group. Data are mean ± s.d. followed by one-way ANOVA with a *post-hoc* test.

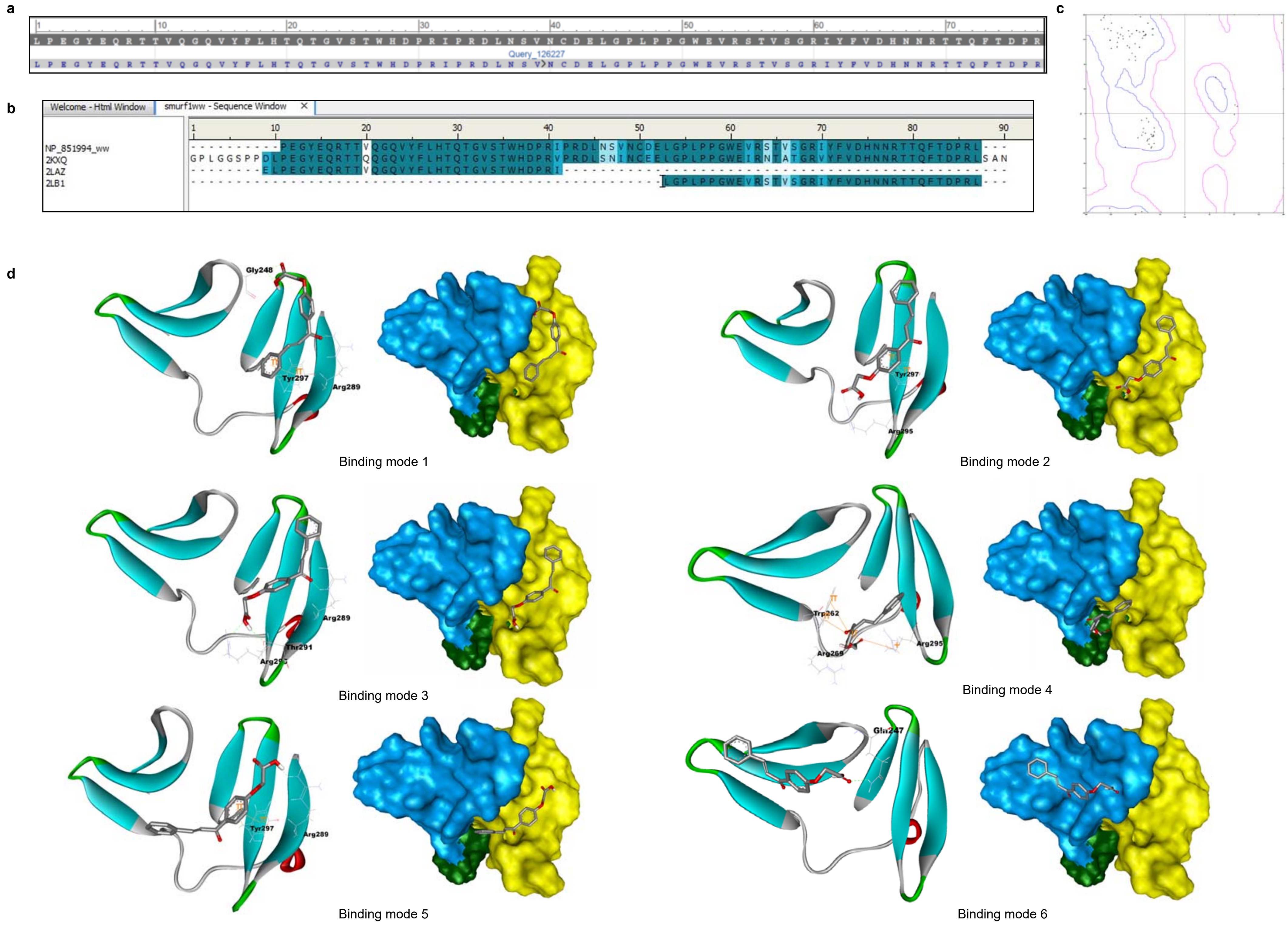

a
LPEGYEQRTTVQGQVYFLHTQTGVSTWHDPRIPRDLNSVNCDELGPLPPGWEVRSTVSGRIYFVDHNNRTTQFTDPR
Query_126227
b
Welcome - Html Window
smurf1ww - Sequence Window
NP_851994_ww
2KXQ
2LAZ
2LB1
c
d
Gly248
Tyr297
Arg289
Binding mode 1
Arg295
Binding mode 2
Thr291
Binding mode 3
Trp262
Arg269
Binding mode 4
Binding mode 5
Gln247
Binding mode 6

Supplementary Figure 7. Predicted binding modes and key residues for interaction between the chalcone derivative and Smurf1 WW1-WW2 domains. (a) Sequence alignments between human and mouse Smurf1 WW1-WW2 domains (upper, human; lower, mouse). **(b)** Multiple sequence alignments between Smurf1 WW1-WW2 and templates. Protein structures of Smurf2 (PDB code: 2KXQ [https://www.rcsb.org/structure/2KXQ]; identity: 86%), Smurf1 WW1 domain (PDB code: 2LAZ [https://www.rcsb.org/structure/2laz]) and Smurf1 WW2 domain (PDB code: 2LB1 [https://www.rcsb.org/structure/2lb1]) were used as templates. **(c)** Ramachandran plot of predicted structure of Smurf1 WW1-WW2. **(d)** 6 top-ranked binding modes with low binding energies generated by AutoDock Vina and key residues for interaction between the chalcone derivative and Smurf1 WW1-WW2 domains. For ribbon forms of the binding modes (left), the beta sheet on the left side was WW1 and the beta sheet on the right side was WW2. Linker region between WW1 and WW2 domains was shown between the two sheets. For surface views of the binding modes (right), the WW1 domain was colored in blue and the WW2 domain was colored with yellow. The linker region between WW1 and WW2 domains was colored with dark green.

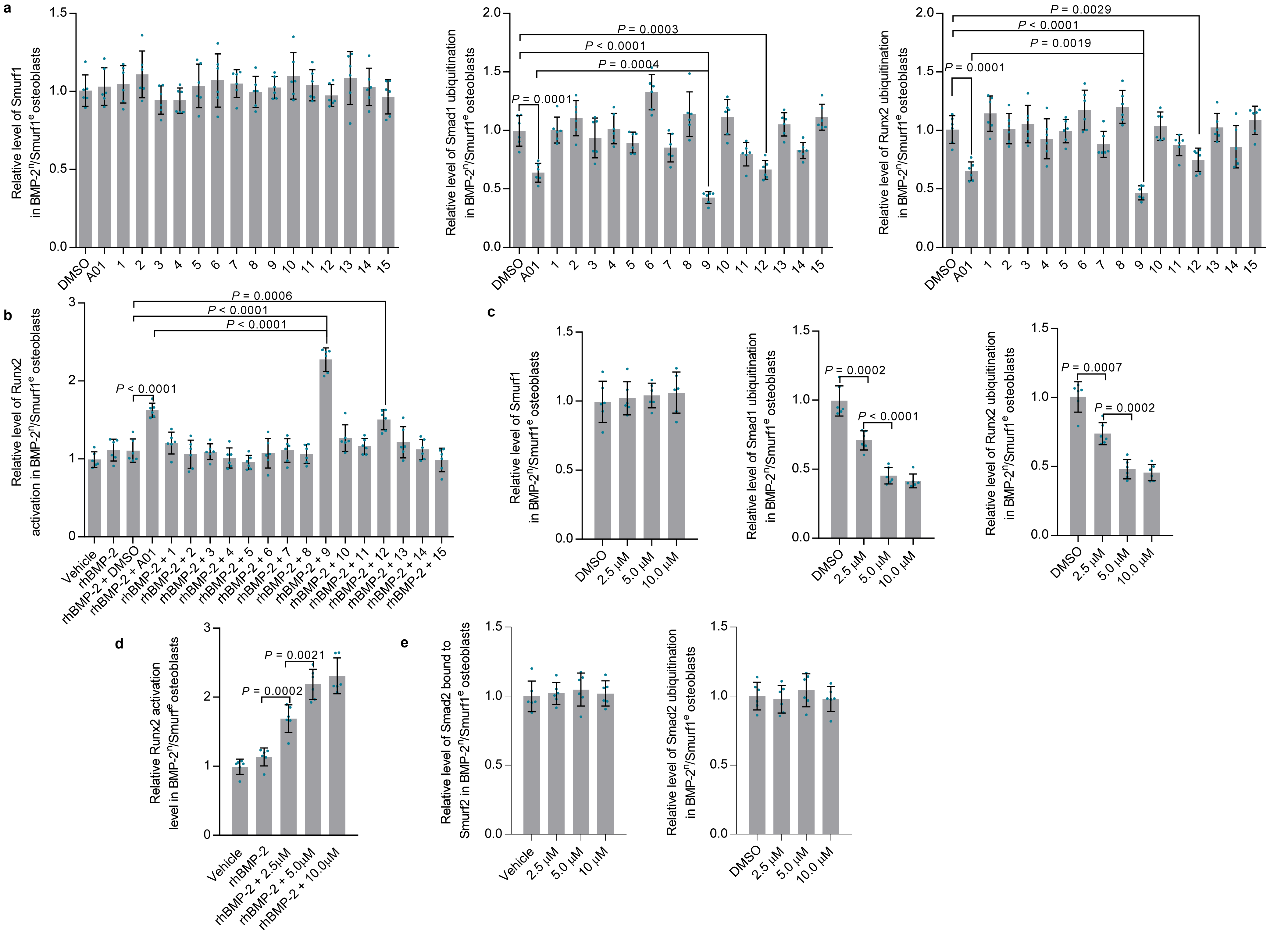
a
Relative level of Smurf1 in BMP-2n/Smurf1e osteoblasts
DMSO
A01
1
2
3
4
5
6
7
8
9
10
11
12
13
14
15
Relative level of Smad1 ubiquitination in BMP-2n/Smurf1e osteoblasts
P = 0.0003
P < 0.0001
P = 0.0004
P = 0.0001
Relative level of Runx2 ubiquitination in BMP-2n/Smurf1e osteoblasts
P = 0.0029
P < 0.0001
P = 0.0019
P = 0.0001
b
Relative level of Runx2 activation in BMP-2n/Smurf1e osteoblasts
P = 0.0006
P < 0.0001
P < 0.0001
P < 0.0001
Vehicle
rhBMP-2
rhBMP-2 + DMSO
rhBMP-2 + A01
rhBMP-2 + 1
rhBMP-2 + 2
rhBMP-2 + 3
rhBMP-2 + 4
rhBMP-2 + 5
rhBMP-2 + 6
rhBMP-2 + 7
rhBMP-2 + 8
rhBMP-2 + 9
rhBMP-2 + 10
rhBMP-2 + 11
rhBMP-2 + 12
rhBMP-2 + 13
rhBMP-2 + 14
rhBMP-2 + 15
c
Relative level of Smurf1 in BMP-2n/Smurf1e osteoblasts
Relative level of Smad1 ubiquitination in BMP-2n/Smurf1e osteoblasts
P = 0.0002
P < 0.0001
Relative level of Runx2 ubiquitination in BMP-2n/Smurf1e osteoblasts
P = 0.0007
P = 0.0002
DMSO
2.5 μM
5.0 μM
10.0 μM
d
Relative Runx2 activation level in BMP-2n/Smurfe osteoblasts
P = 0.0021
P = 0.0002
Vehicle
rhBMP-2
rhBMP-2 + 2.5μM
rhBMP-2 + 5.0μM
rhBMP-2 + 10.0μM
e
Relative level of Smad2 bound to Smurf2 in BMP-2n/Smurf1e osteoblasts
Vehicle
2.5 μM
5.0 μM
10 μM
Relative level of Smad2 ubiquitination in BMP-2n/Smurf1e osteoblasts
DMSO
2.5 μM
5.0 μM
10.0 μM

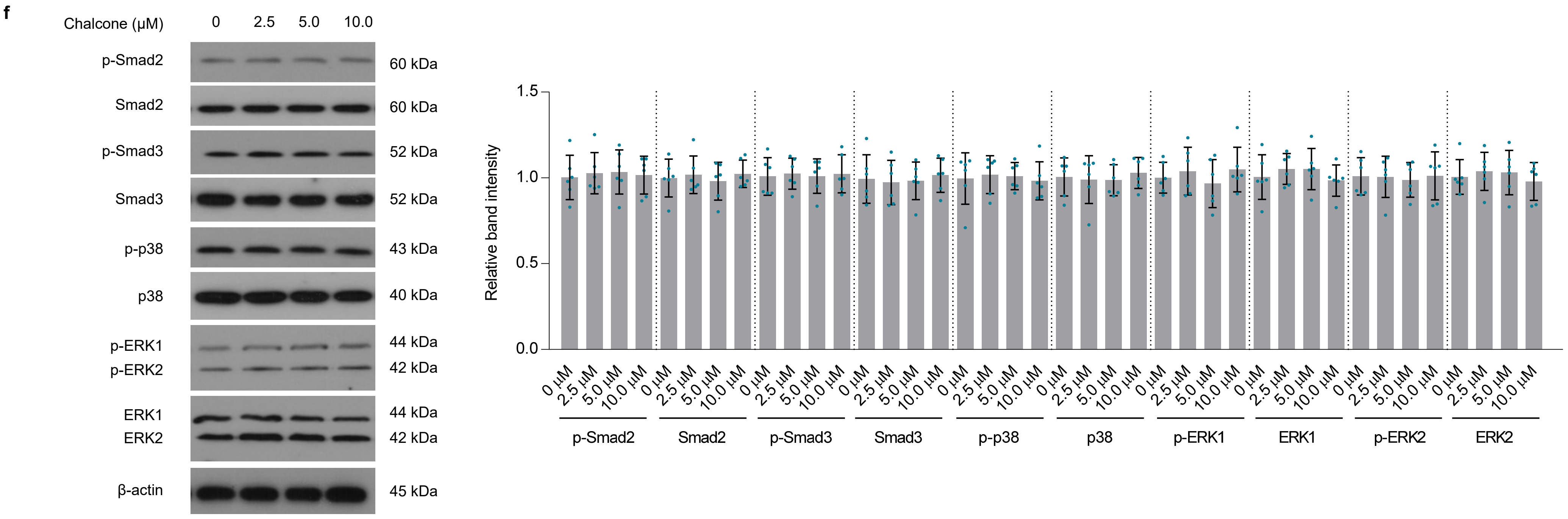

Supplementary Figure 8. Effects of small molecules on Smurf1/2 activity, BMP/TGF-β signaling and osteogenic differentiation in osteoblasts from mice BMP-2ⁿ/Smurf1ᵉ subgroup. (a) Effects of the top-ranked 15 small molecules (5.0 μM) selected by molecular docking-based virtual screening on Smurf1 expression and Smurf1 activity (ubiquitination of Smad1 and Runx2) in osteoblasts from BMP-2ⁿ/Smurf1ᵉ subgroup of 15-month-old osteoporotic mice (OVX at 6 months old). A01, a previously reported Smurf1 inhibitor. **(b)** Effects of the top-ranked 15 small molecules (5.0 μM) on Runx2 activation in osteoblasts (from BMP-2ⁿ/Smurf1ᵉ subgroup) with presence of 100 ng ml^{-1} rhBMP-2 or vehicle (PBS). **(c)** Relative level of Smurf1 expression and Smurf1 activity (ubiquitination of Smad1 and Runx2) in osteoblasts (from BMP-2ⁿ/Smurf1ᵉ subgroup) incubated with the chalcone derivative at a series of concentrations (2.5, 5.0 and 10.0 μM). **(d)** Relative level of Runx2 activation in osteoblasts (from BMP-2ⁿ/Smurf1ᵉ subgroup) incubated with the chalcone derivative at a series of concentrations (2.5, 5.0 and 10.0 μM), with the presence of 100 ng ml^{-1} rhBMP-2 or vehicle (PBS). **(e)** Relative level of Smurf2 activity (Smad2 bound to Smurf2 and ubiquitination of Smad2) in osteoblasts (from BMP-2ⁿ/Smurf1ᵉ subgroup) incubated with the chalcone derivative at a series of concentrations (2.5, 5.0 and 10.0 μM). **(f)** Levels of expression and activation (phosphorylation) of downstream molecules of TGF-βs including Smad2/3, ERK1/2 and p38 in osteoblasts (from BMP-2ⁿ/Smurf1ᵉ subgroup) incubated with the chalcone derivative at a series of concentrations (0, 2.5, 5.0 and 10.0 μM). Left, representative images of western blots; right, relative band intensity normalized to internal reference β-actin. The levels of Smurf1 expression, ubiquitination of Smad1, Smad2 and Runx2, Smad2 bound to Smurf2, Runx2 activation and osteocalcin mRNA were normalized to the mean values of osteoblasts treated with vehicle (PBS) or DMSO. *n* = 6 per group. Data are mean ± s.d. followed by one-way ANOVA with a *post-hoc* test.

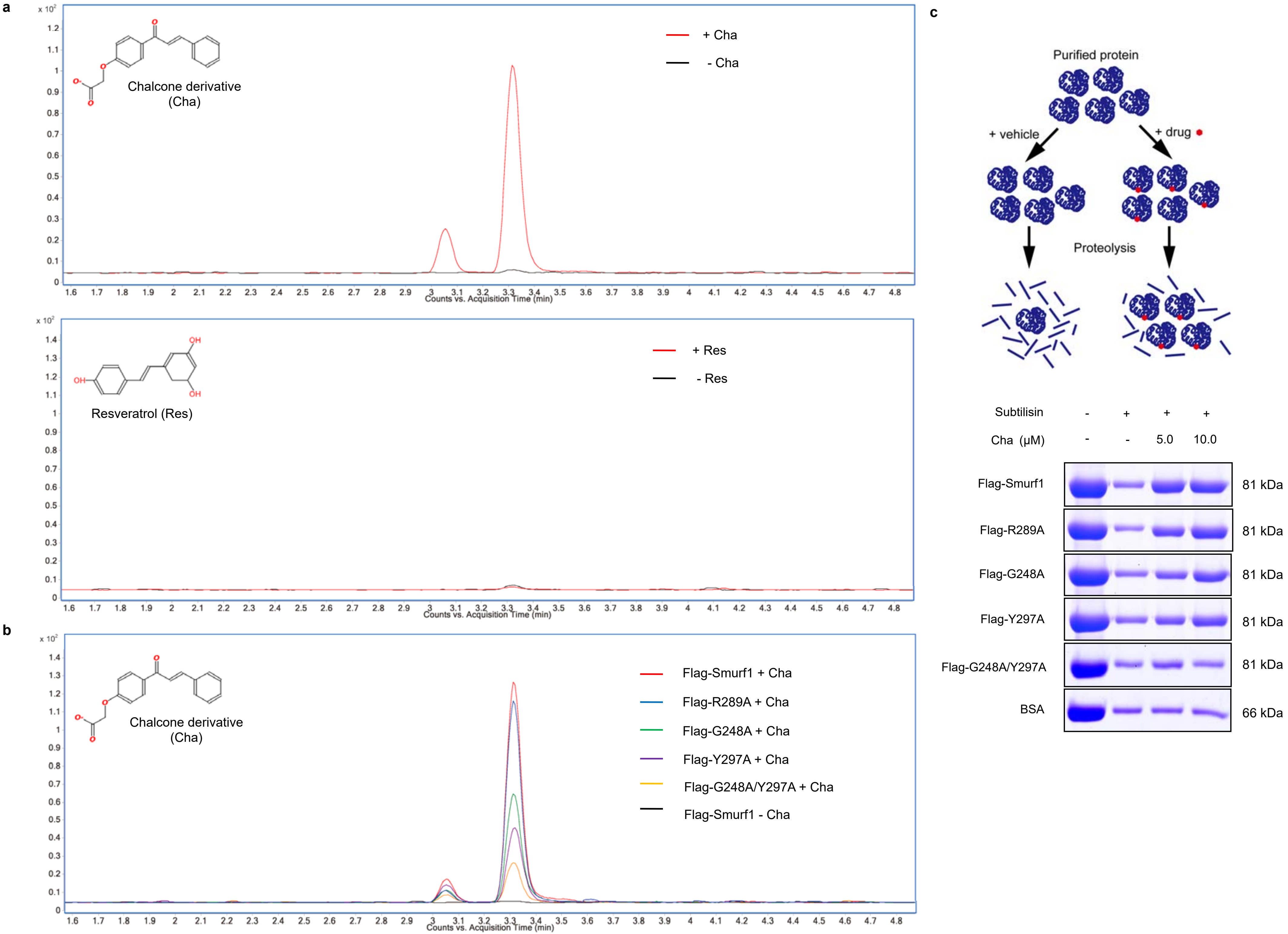
a
x 10²
Chalcone derivative
(Cha)
+ Cha
- Cha
Counts vs. Acquisition Time (min)
x 10²
Resveratrol (Res)
+ Res
- Res
Counts vs. Acquisition Time (min)
b
x 10²
Chalcone derivative
(Cha)
Flag-Smurf1 + Cha
Flag-R289A + Cha
Flag-G248A + Cha
Flag-Y297A + Cha
Flag-G248A/Y297A + Cha
Flag-Smurf1 - Cha
Counts vs. Acquisition Time (min)
c
Purified protein
+ vehicle
+ drug
Proteolysis
Subtilisin - + + +
Cha (μM) - - 5.0 10.0
Flag-Smurf1 81 kDa
Flag-R289A 81 kDa
Flag-G248A 81 kDa
Flag-Y297A 81 kDa
Flag-G248A/Y297A 81 kDa
BSA 66 kDa

Supplementary Figure 9. Validation of the interaction between the chalcone derivative and Smurf1. (a) Immunoprecipitation using anti-Smurf1 antibody to examine the interaction between Smurf1 and the chalcone derivative (upper) or resveratrol (negative control; lower) in osteoblasts from BMP-2^{n}/Smurf1^{e} subgroup of 15-month-old osteoporotic mice (OVX at 6 months old). **(b)** Immunoprecipitation using anti-Flag antibody to examine the interaction between the chalcone derivative and Flag-Smurf1 wild type or mutants (R289A, G248A, Y297A and G248A/Y297A) in osteoblasts from BMP-2^{n}/Smurf1^{e} subgroup. The cells were transfected with pcDNA3.1-based expression vectors for Flag-Smurf1 wild type or mutants and then incubated with the chalcone derivative, resveratrol or vehicle (DMSO) before immunoprecipitation. The chalcone derivative or resveratrol in immunoprecipitates was examined by LC-MS/MS. **(c)** Drug affinity responsive target stability (DARTS) assay to determine binding of the chalcone derivative to Smurf1 wild type or mutants. DARTS was performed by simply treating purified Flag-Smurf1 wild type, mutants or BSA with the chalcone derivative (5.0 and 10.0 μM) or vehicle (DMSO), followed by digestion of the proteins with proteases (subtilisin) (upper). Subsequently, the samples are separated by sodium dodecyl sulfate polyacrylamide gel electrophoresis (SDS-PAGE) and stained by coomassie brilliant blue to identify protein bands that were protected from proteolysis by the chalcone derivative (lower). Cha: the chalcone derivative; Res: resveratrol.

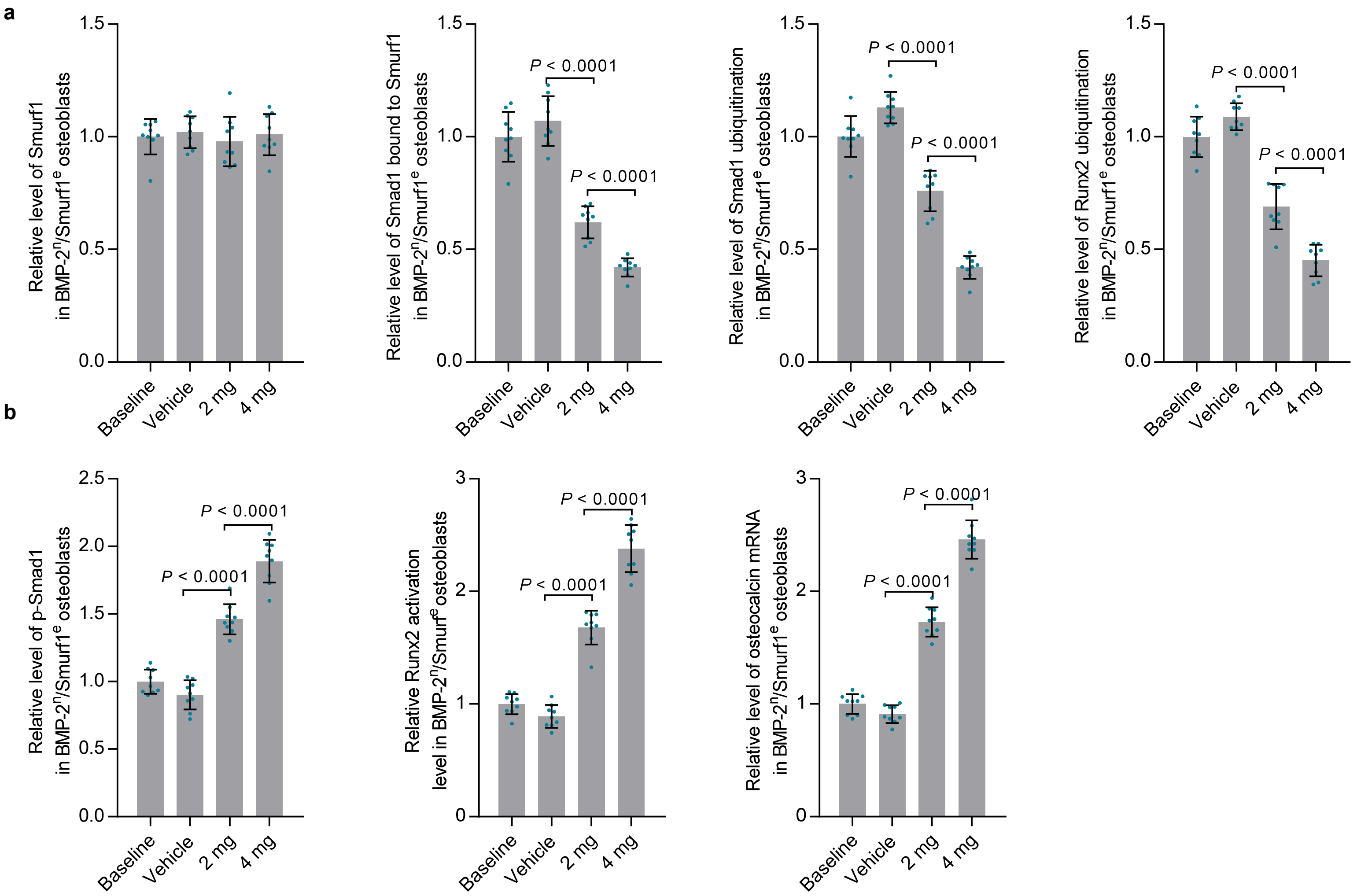

Supplementary Figure 10. Effects of chalcone derivative on Smurf1 activity, BMP signaling and osteogenic potential in osteoblasts from mice BMP-2^{n}/Smurf1^{e} subgroup during spinal fusion. (a) Relative levels of Smurf1 expression and Smurf1 activity (Smad1 bound to Smurf1, ubiquitination of Smad1 and Runx2) in osteoblasts isolated from spinal fusion sites (L4-L6 vertebrae) in BMP-2^{n}/Smurf1^{e} subgroup of 15-month-old osteoporotic mice (OVX at 6 months old), administrated with the chalcone derivative (2 mg or 4 mg) or vehicle (DMSO). **(b)** Relative levels of p-Smad1, Runx2 activation and osteocalcin mRNA in the above osteoblasts. Relative levels of Smurf1 and p-Smad1, Smad1 bound to Smurf1, Runx2 activation, ubiquitination of Smad1 and Runx2 and osteocalcin mRNA were normalized to the mean values of osteoblasts from BMP-2^{n}/Smurf1^{e} subgroup before the spinal fusion surgery (baseline). n = 9 per group. Data are mean ± s.d. followed by one-way ANOVA with a *post-hoc* test.

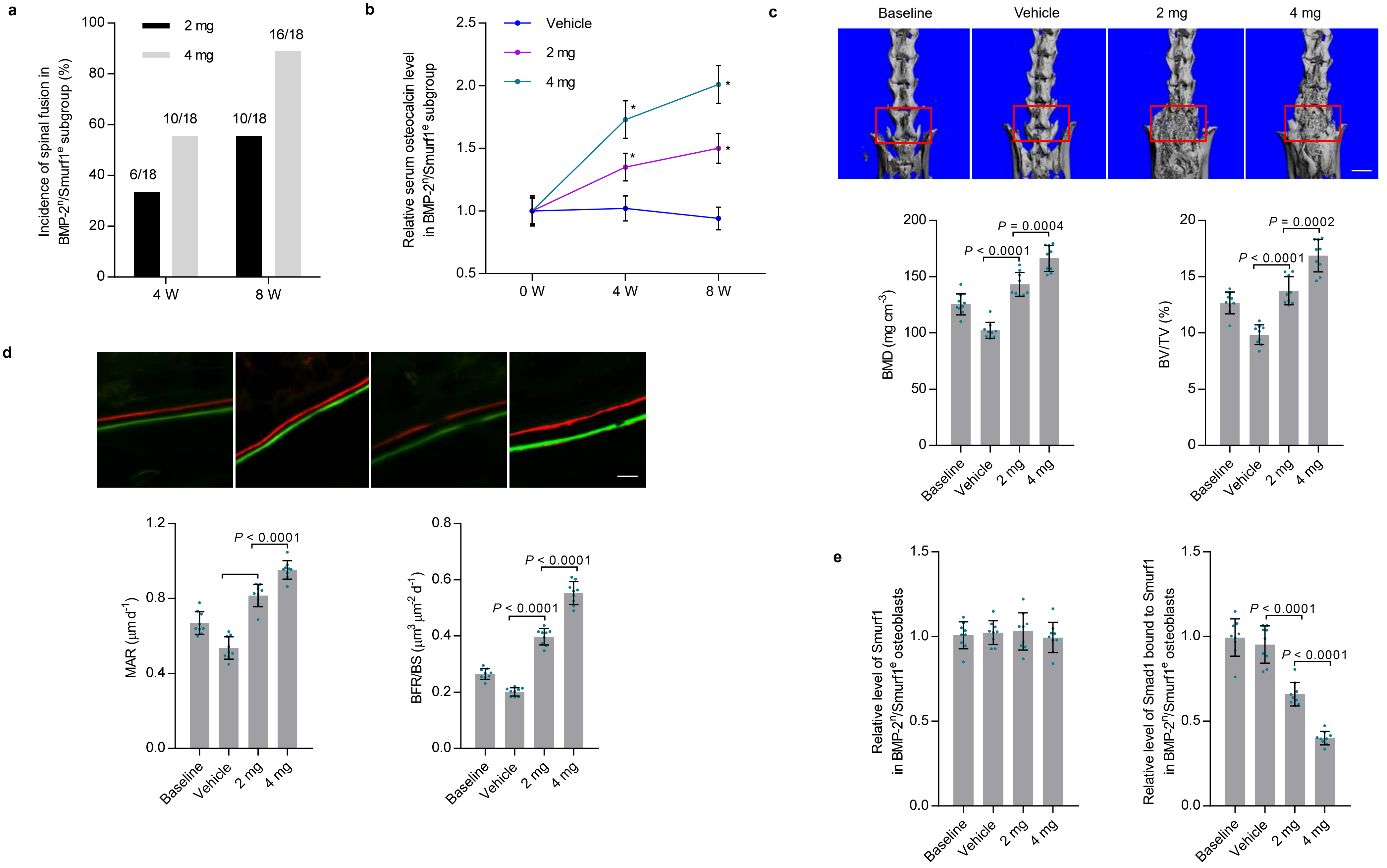

a
Incidence of spinal fusion in BMP-2n/Smurf1e subgroup (%)
2 mg
4 mg
6/18
10/18
10/18
16/18
4 W
8 W
b
Relative serum osteocalcin level in BMP-2n/Smurf1e subgroup
Vehicle
2 mg
4 mg
0 W
4 W
8 W
c
Baseline
Vehicle
2 mg
4 mg
BMD (mg cm-3)
P < 0.0001
P = 0.0004
BV/TV (%)
P < 0.0001
P = 0.0002
d
MAR (μm d-1)
P < 0.0001
BFR/BS (μm3 μm-2 d-1)
P < 0.0001
P < 0.0001
e
Relative level of Smurf1 in BMP-2n/Smurf1e osteoblasts
Relative level of Smad1 bound to Smurf1 in BMP-2n/Smurf1e osteoblasts
P < 0.0001
P < 0.0001

f

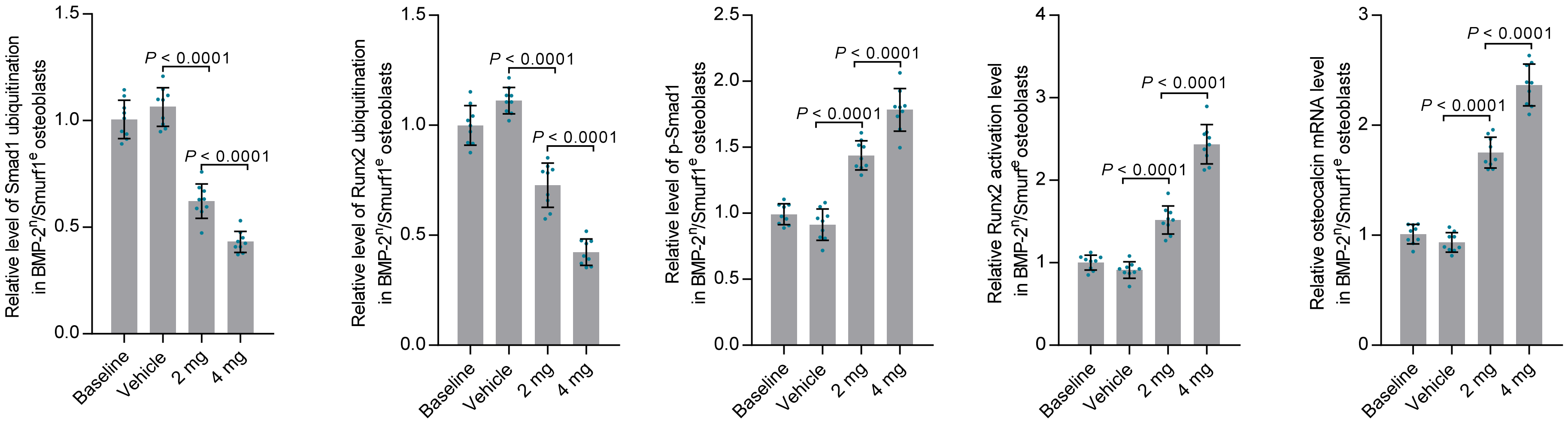

Supplementary Figure 11. Effects of the chalcone derivative on local bone formation, Smurf1 activity and BMP signaling in mice BMP-2^{n}/Smurf1^{e} subgroup during spinal fusion. (a) Manual assessment of spinal fusion incidence during local administration of the chalcone derivative (2 or 4 mg per piece of ACS bilaterally) in BMP-2^{n}/Smurf1^{e} subgroup of 8-month-old osteoporotic mice (OVX at 6 months old) at 4 weeks (4 W) or 8 weeks (8 W). n = 18 per group. Fisher's exact test was performed (P = 0.007 and P < 0.0001 for 2 mg and 4 mg versus vehicle at 4 W, respectively; P < 0.0001 for 2 mg or 4 mg versus vehicle at 8 W). **(b)** Relative serum osteocalcin level during local administration of the chalcone derivative or vehicle (DMSO) in mice BMP-2^{n}/Smurf1^{e} subgroup. The level of serum osteocalcin in BMP-2^{n}/Smurf1^{e} subgroup was normalized to the baseline before spinal fusion surgery. *P < 0.0001 for 2 mg versus vehicle or 4 mg versus 2 mg at 4 W and 8 W. **(c)** Representative microCT images (upper) and bone mass parameters (BMD and BV/TV) (bottom) in spinal fusion sites (indicated by red rectangle). Scale bars, 2.5 mm. **(d)** Representative images showing bone formation in spinal fusion sites assessed by xylenol (red) and calcein (green) labeling and analysis of dynamic bone histomorphometric parameters (MAR and BFR/BS). Scale bars, 10 μm. **(e)** Relative levels of Smurf1 expression and Smurf1 activity (Smad1 bound to Smurf1) in osteoblasts isolated from spinal fusion sites (L4-L6 vertebrae) in BMP-2^{n}/Smurf1^{e} subgroup of aged osteoporotic mice administrated with the chalcone derivative (2 mg or 4 mg) or vehicle (DMSO). **(f)** Relative levels of p-Smad1, Smurf1 activity (ubiquitination of Smad1 and Runx2), Runx2 activation and osteocalcin mRNA in osteoblasts isolated from spinal fusion sites (L4-L6 vertebrae) in BMP-2^{n}/Smurf1^{e} subgroup administrated with the chalcone derivative (2 mg or 4 mg) or vehicle (DMSO). Relative levels of Smurf1 and p-Smad1, Smad1 bound to Smurf1, Runx2 activation, ubiquitination of Smad1 and Runx2 and osteocalcin mRNA were normalized to the mean values of baseline before the spinal fusion surgery. n = 9 per group. Data are mean ± s.d. followed by one-way ANOVA with a *post-hoc* test.

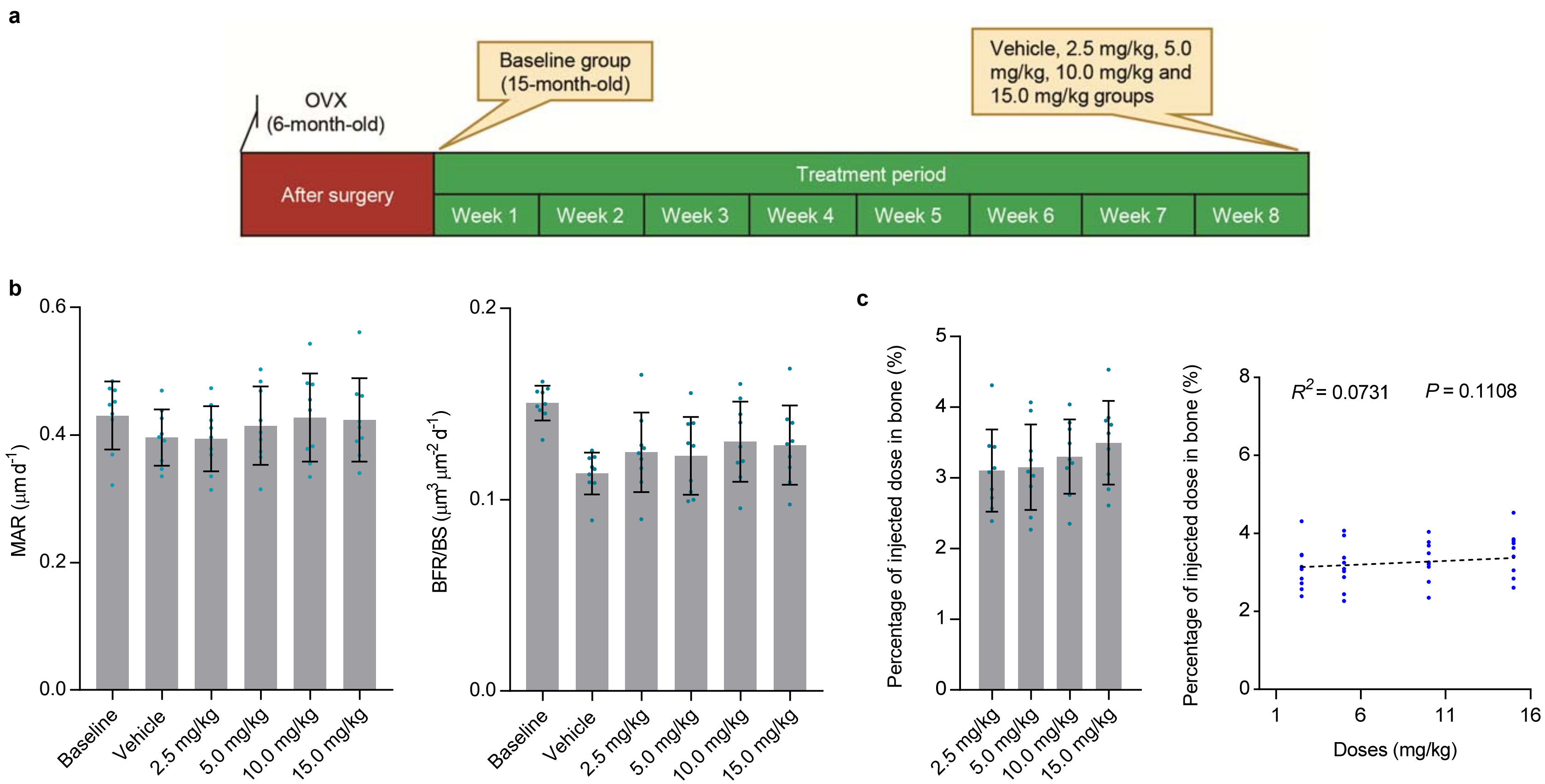

Supplementary Figure 12. Systemic bone anabolic action and intraosseous accumulation of the chalcone derivative in BMP-2^n/Smurf1^e mice. (a) A schematic diagram illustrating the experimental design. **(b)** Analysis of dynamic bone histomorphometric parameters (MAR and BFR/BS) at the proximal tibia in BMP-2^n/Smurf1^e subgroup of 15-month-old osteoporotic mice (OVX at 6 months old) with intravenously administration of the chalcone derivative at a series of doses (2.5, 5.0, 10.0 and 15.0 mg kg^{-1}) or vehicle (DMSO). **(c)** Intraosseous accumulation of the chalcone derivative (left) and correlation analysis of Intraosseous accumulation of the chalcone derivative with the injected doses (right). Amount of the chalcone derivative in bone tissues was normalized to the injected dose. *n* = 9 per group. Data are mean ± s.d. followed by one-way ANOVA with a *post-hoc* test.

Model Summary

Model	R	R Square	Adjusted R Square	Std. Error of the Estimate
1	0.771[a]	0.595	0.571	0.039872

a. Predictors: (Constant), Accumulation, Dose

ANOVA[a]

Model		Sum of Squares	df	Mean Square	F	Sig.
1	Regression	0.077	2	0.039	24.246	0.000[b]
	Residual	0.052	33	0.002		
	Total	0.130	35			

a. Dependent Variable: MAR

b. Predictors: (Constant), Accumulation, Dose

Coefficients[a]

Model		Unstandardized Coefficients		Standardized Coefficients	t	Sig.
		B	Std. Error	Beta		
1	(Constant)	0.148	0.039		3.785	0.001
	Dose	0.000	0.001	-0.036	-0.311	0.758
	Accumulation	0.083	0.012	0.780	6.782	0.000

a. Dependent Variable: MAR

Supplementary Figure 13. Association of the injected doses or bone accumulation of the chalcone derivative with MAR. A multiple linear regression analysis was used to determine the association of the injected doses or bone accumulation of the chalcone derivative with MAR in BMP-2^{n}/Smurf1^{e} subgroup of 15-month-old osteoporotic mice (OVX at 6-months old) intravenously administered with the chalcone derivative at a series of doses (2.5, 5.0, 10.0 and 15.0 mg kg^{-1}). Dependent Variable: MAR; Predictors: bone accumulation and injected doses of the chalcone derivative.

a

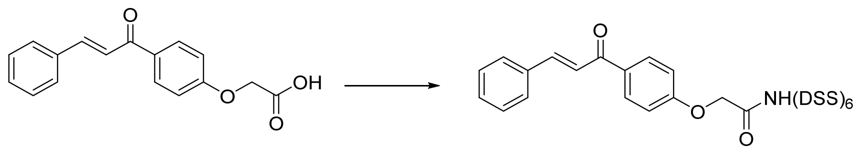

b

Measurement:	Peak Area	Run Time:	10min
Calculation Type:	Percent	Wavelength :	220nm
Flow Rate :	1ml/min	Inj.Vol:	10uL
Buffer A :	0.1% TFA in water	Buffer B:	0.1%TFA in Acetonitrile
Column:	Kromasil 100-5C18,4.6mmX250mm,5 micron	Column Temp:	25℃
Gradient(linear):	35%-60% buffer B in 10min		

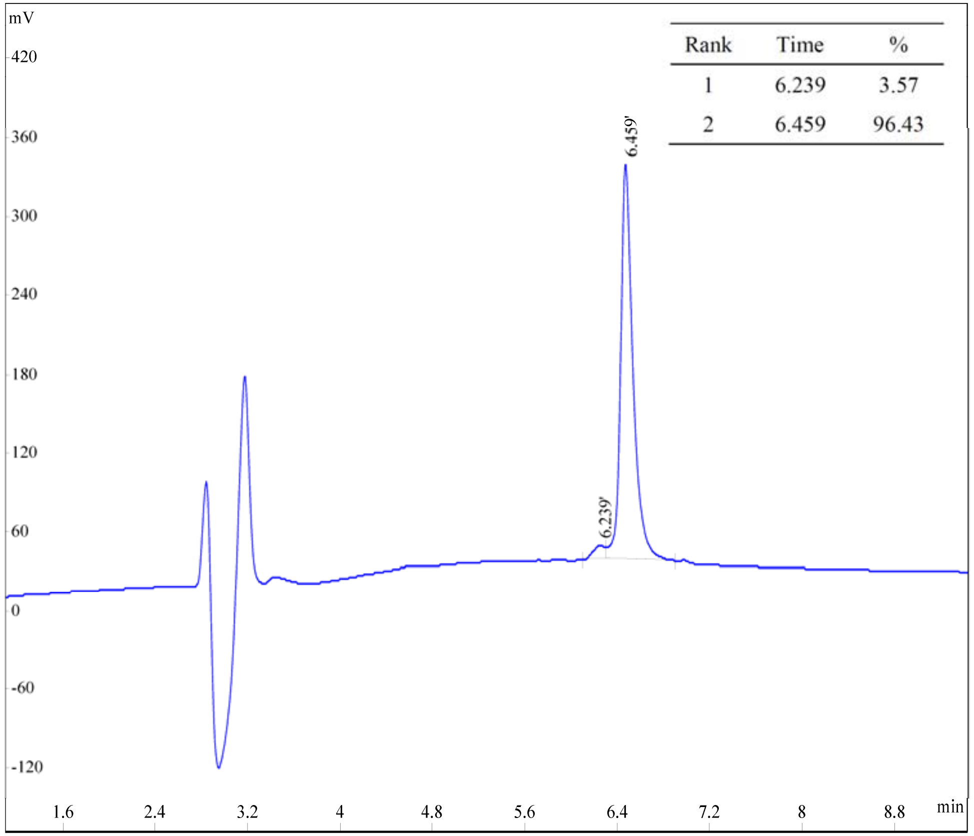

c

Expected MS: 2017.59		Mass Spectrometer: API 150EX	
Ion Source: ESI		B.conc: 75%ACN/24.5%H_2O/0.5%Ac	
NEB:10.00	CUR:12.00	IS:-4500	TEM:0.00
Flow Rate : 0.2ml/min		Run Time: 1min	

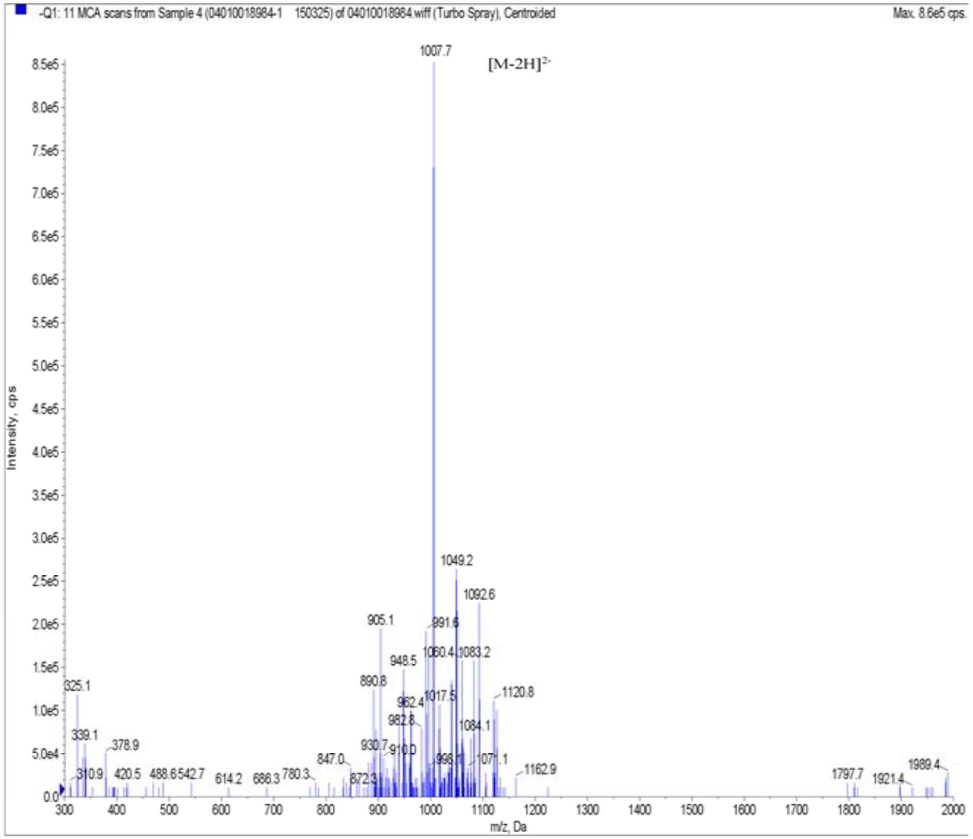

Supplementary Figure 14. Synthesis and identification of $(DSS)_6$-chalcone derivative conjugate. (a) Synthesis of $(DSS)_6$-chalcone derivative conjugate by an amide bond formed via a reaction between the carboxyl group of the chalcone derivative and the amino group of $(DSS)_6$. $(DSS)_6$ are six repetitive sequences of aspartate-serine-serine. **(b)** Identification of $(DSS)_6$-chalcone derivative conjugate by reverse phase high performance liquid chromatography (RP-HPLC). The retention time was 6.459 min and the purity were 96.43%. **(c)** Identification of $(DSS)_6$-chalcone derivative conjugate by electrospray ionisation mass spectrometry (ESI-MS). The MS determined the m/z value, that was the molecular weight divided by the charge. Due to the formation of $[M-2H]^{2-}$ ions, the detected molecule weight (1007.7) was half of the real molecular weight, which was consistent with the expected molecule weight (2017.59).

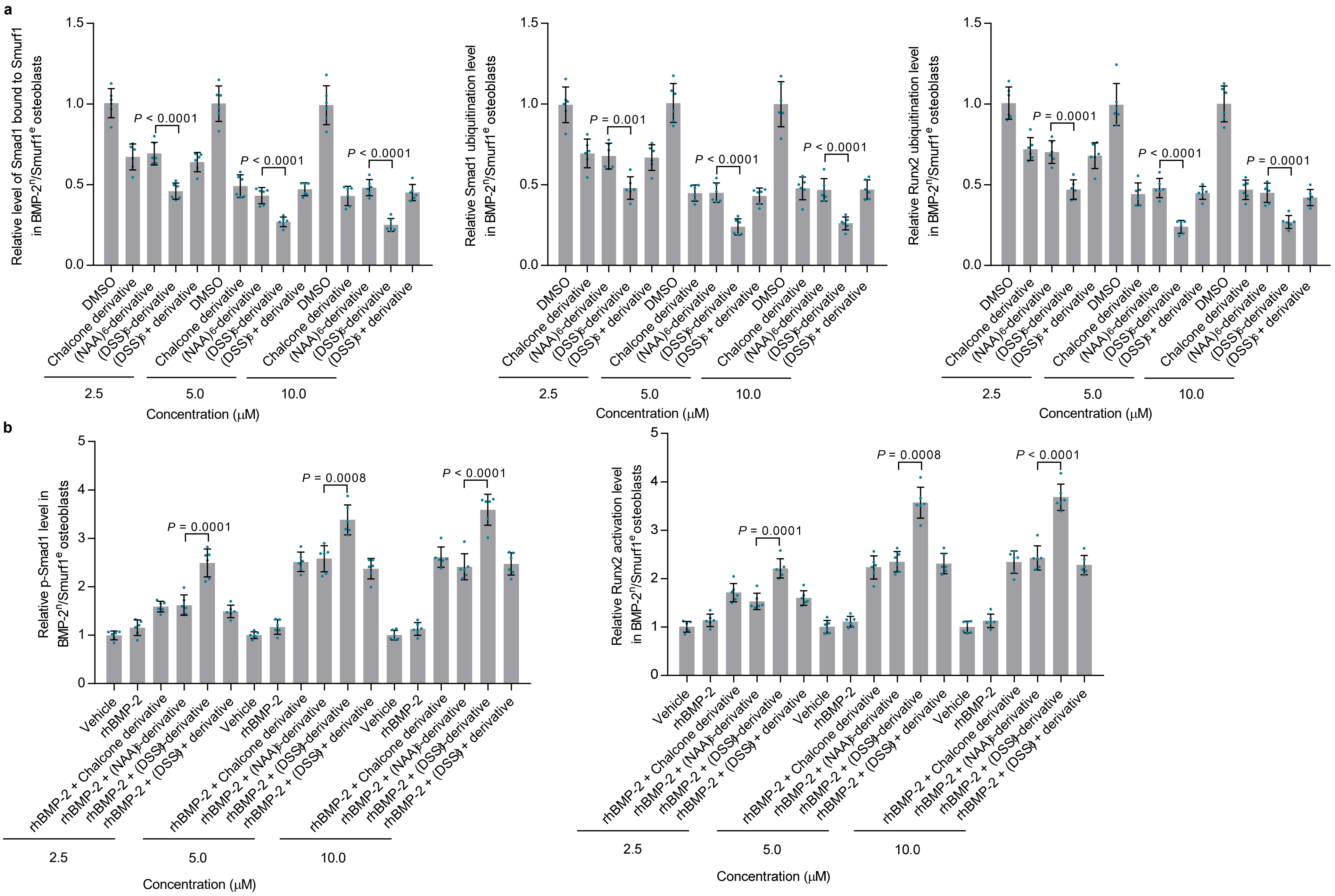

a
Relative level of Smad1 bound to Smurf1 in BMP-2ⁿ/Smurf1ᵉ osteoblasts
P < 0.0001
P < 0.0001
P < 0.0001
Relative Smad1 ubiquitination level in BMP-2ⁿ/Smurf1ᵉ osteoblasts
P = 0.001
P < 0.0001
P < 0.0001
Relative Runx2 ubiquitination level in BMP-2ⁿ/Smurf1ᵉ osteoblasts
P = 0.0001
P < 0.0001
P = 0.0001
1.5
1.0
0.5
0.0
DMSO
Chalcone derivative
(NAA)6-derivative
(DSS)6-derivative
(DSS)6 + derivative
2.5
5.0
10.0
Concentration (μM)
b
Relative p-Smad1 level in BMP-2ⁿ/Smurf1ᵉ osteoblasts
P = 0.0001
P = 0.0008
P < 0.0001
Relative Runx2 activation level in BMP-2ⁿ/Smurf1ᵉ osteoblasts
P = 0.0001
P = 0.0008
P < 0.0001
5
4
3
2
1
0
Vehicle
rhBMP-2
rhBMP-2 + Chalcone derivative
rhBMP-2 + (NAA)6-derivative
rhBMP-2 + (DSS)6-derivative
rhBMP-2 + (DSS)6 + derivative
2.5
5.0
10.0
Concentration (μM)

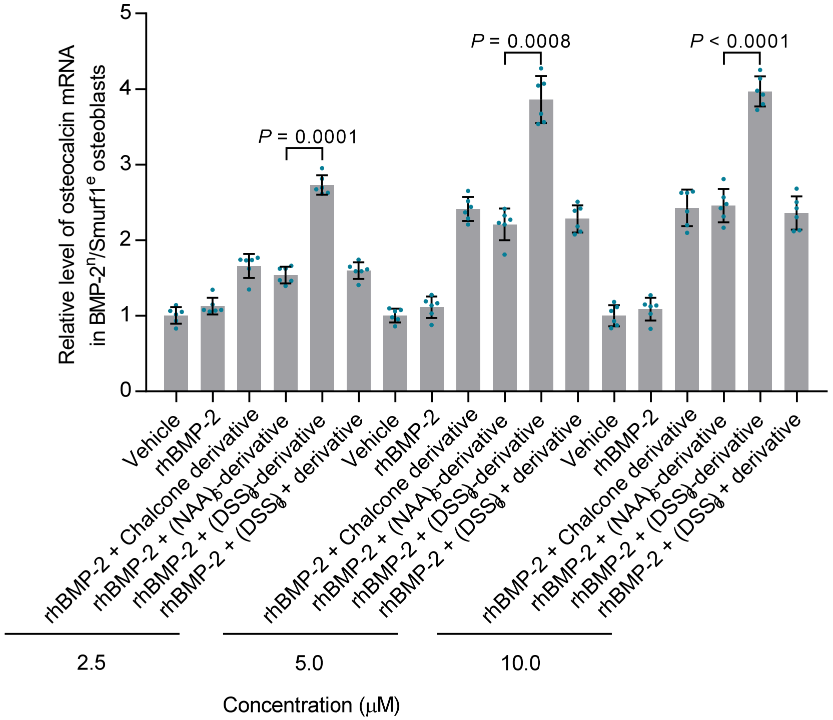
Relative level of osteocalcin mRNA in BMP-2n/Smurf1e osteoblasts
P = 0.0001
P = 0.0008
P < 0.0001
Vehicle
rhBMP-2
rhBMP-2 + Chalcone derivative
rhBMP-2 + (NAA)6-derivative
rhBMP-2 + (DSS)6-derivative
rhBMP-2 + (DSS)6 + derivative
2.5
5.0
10.0
Concentration (μM)

c

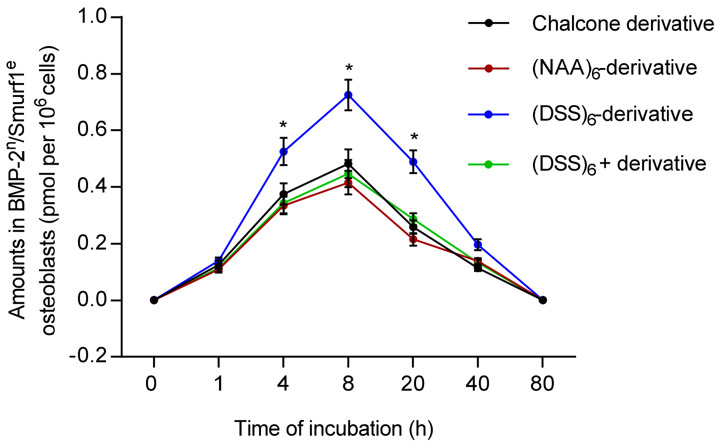
Amounts in BMP-2n/Smurf1e osteoblasts (pmol per 10^6 cells)
Time of incubation (h)
Chalcone derivative
(NAA)6-derivative
(DSS)6-derivative
(DSS)6 + derivative

d

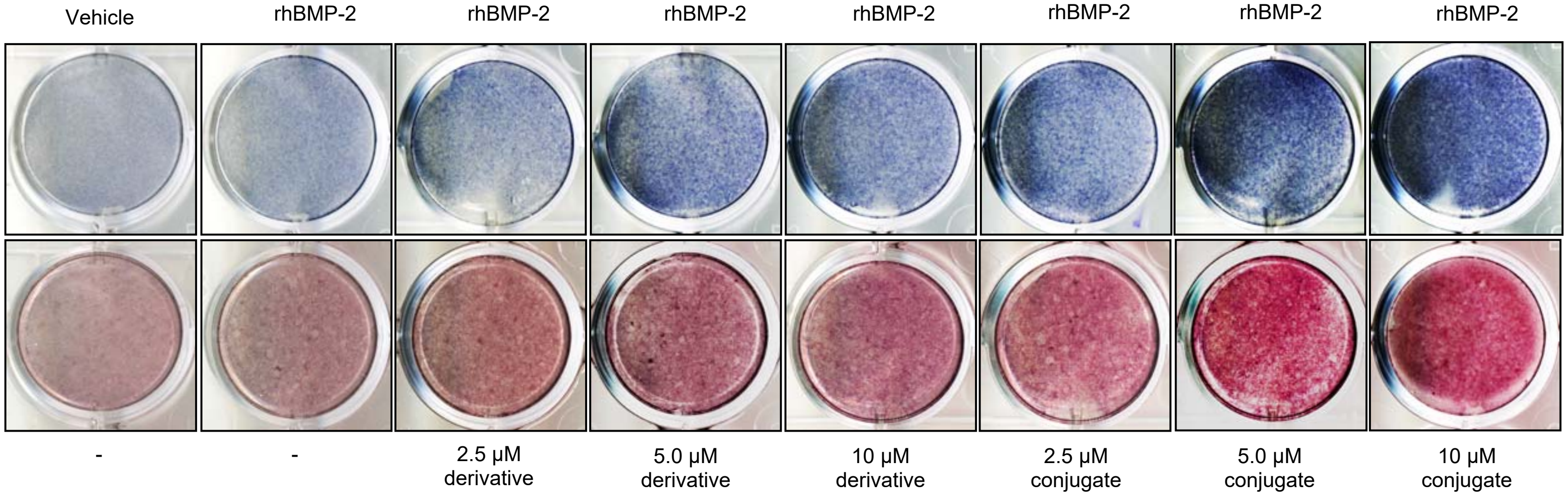
Vehicle
rhBMP-2
rhBMP-2
rhBMP-2
rhBMP-2
rhBMP-2
rhBMP-2
rhBMP-2
-
-
2.5 μM derivative
5.0 μM derivative
10 μM derivative
2.5 μM conjugate
5.0 μM conjugate
10 μM conjugate

e

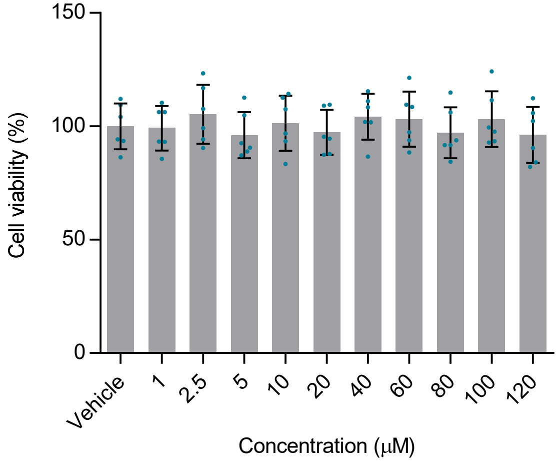
Cell viability (%)
Vehicle
1
2.5
5
10
20
40
60
80
100
120
Concentration (μM)

Supplementary Figure 15. Effects of different chalcone derivative formulations on Smurf1 activity and BMP signaling in osteoblasts from mice BMP-2^{n}/Smurf1^{e} subgroup. (a) Relative level of Smurf1 activity (Smad1 bound to Smurf1, ubiquitination of Smad1 and Runx2) in osteoblasts (from BMP-2^{n}/Smurf1^{e} subgroup of 15-month-old osteoporotic mice) incubated with the chalcone derivative, $(DSS)_6$-derivative, $(NAA)_6$-derivative, $(DSS)_6$ + derivative, at a series of concentrations (2.5, 5.0 and 10.0 μM), respectively. **(b)** Relative level of p-Smad1, Runx2 activation and osteocalcin mRNA in osteoblasts (from BMP-2^{n}/Smurf1^{e} subgroup) incubated with the chalcone derivative, $(DSS)_6$-derivative, $(NAA)_6$-derivative and $(DSS)_6$ + derivative, at a series of concentrations (2.5, 5.0 and 10.0 μM), respectively, with the presence of 100 ng ml^{-1} rnBMP-2. **(c)** Amounts of chalcone derivative or $(DSS)_6$-derivative or $(NAA)_6$-derivative or $(DSS)_6$ + derivative in osteoblasts (from BMP-2^{n}/Smurf1^{e} subgroup) at a series of incubation times (0, 1, 4, 8, 20, 40 and 80 h) determined by LC-MS/MS. *$P < 0.0001$ for $(DSS)_6$-derivative versus $(NAA)_6$-derivative at 4, 8 and 20 h. **(d)** Alp and alizarin red staining showing effects of the chalcone derivative or $(DSS)_6$-derivative on Alp activity and mineralized nodule formation in BMP-2^{n}/Smurf1^{e} osteoblasts treated with rhBMP-2. **(e)** MTT assay showing *in vitro* cell viability of osteoblasts (from BMP-2^{n}/Smurf1^{e} subgroup) incubated with the $(DSS)_6$-derivative at a series of concentrations (1.0, 2.5, 5.0, 10.0, 20.0 and 60.0, 80.0, 100.0 and 120.0 μM). The levels of Smad1 bound to Smurf1, Smurf1 and p-Smad1, ubiquitination of Smad1 and Runx2, Runx2 activation and osteocalcin mRNA were normalized to the mean values of osteoblasts treated with DMSO or vehicle (PBS). $n = 6$ per group. Data are mean ± s.d. followed by one-way ANOVA with a *post-hoc* test.

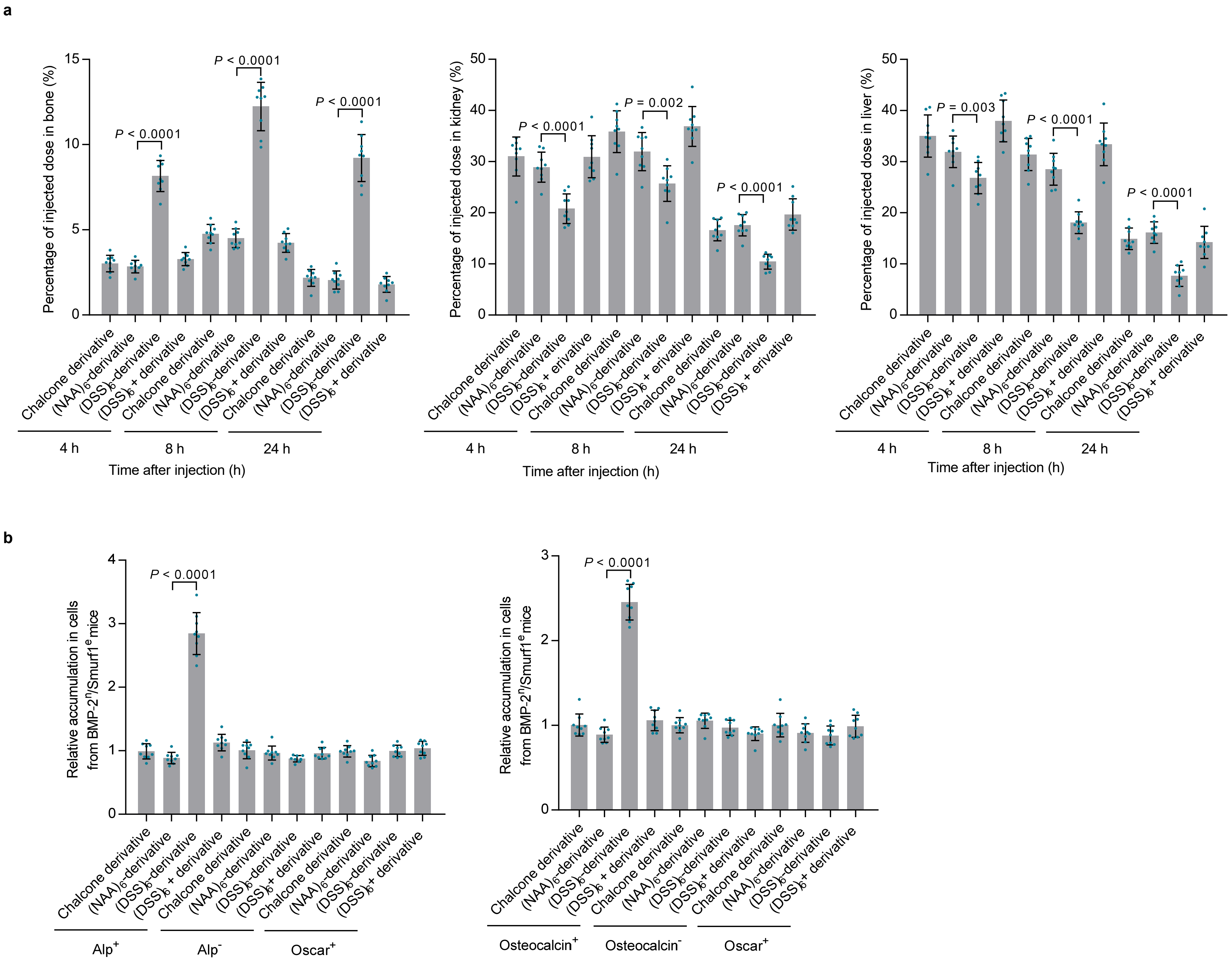
a
Percentage of injected dose in bone (%)
Percentage of injected dose in kidney (%)
Percentage of injected dose in liver (%)
P < 0.0001
P = 0.002
P = 0.003
Chalcone derivative
$(NAA)_6$-derivative
$(DSS)_6$-derivative
$(DSS)_6$ + derivative
$(DSS)_6$ + erivative
4 h
8 h
24 h
Time after injection (h)
b
Relative accumulation in cells from BMP-2^{n}/Smurf1^{e} mice
Alp^{+}
Alp^{-}
Oscar^{+}
Osteocalcin^{+}
Osteocalcin^{-}

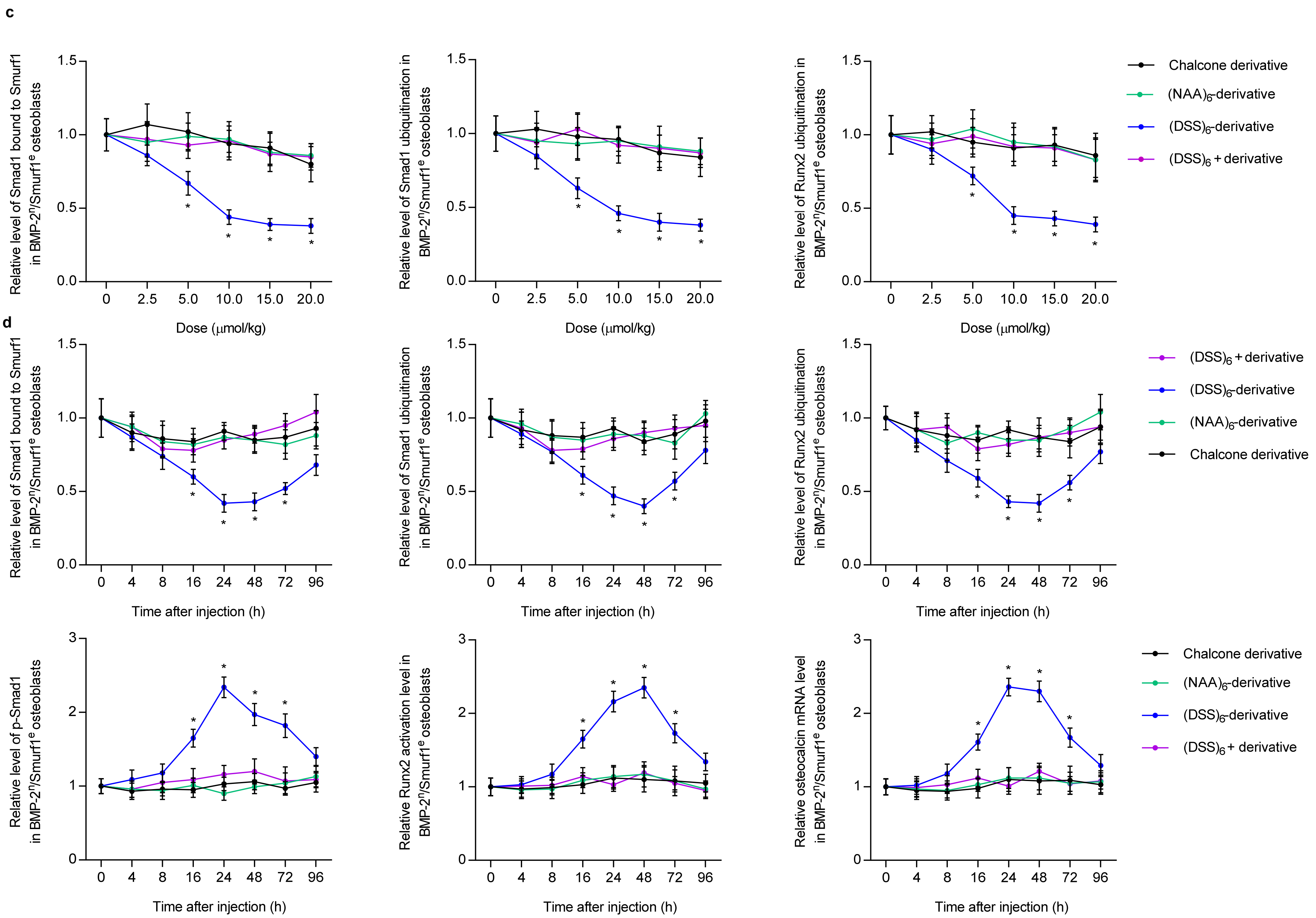
c
Relative level of Smad1 bound to Smurf1 in BMP-2ⁿ/Smurf1ᵉ osteoblasts
Relative level of Smad1 ubiquitination in BMP-2ⁿ/Smurf1ᵉ osteoblasts
Relative level of Runx2 ubiquitination in BMP-2ⁿ/Smurf1ᵉ osteoblasts
Dose (μmol/kg)
0 2.5 5.0 10.0 15.0 20.0
Chalcone derivative
(NAA)6-derivative
(DSS)6-derivative
(DSS)6 + derivative
d
Relative level of Smad1 bound to Smurf1 in BMP-2ⁿ/Smurf1ᵉ osteoblasts
Relative level of Smad1 ubiquitination in BMP-2ⁿ/Smurf1ᵉ osteoblasts
Relative level of Runx2 ubiquitination in BMP-2ⁿ/Smurf1ᵉ osteoblasts
Relative level of p-Smad1 in BMP-2ⁿ/Smurf1ᵉ osteoblasts
Relative Runx2 activation level in BMP-2ⁿ/Smurf1ᵉ osteoblasts
Relative osteocalcin mRNA level in BMP-2ⁿ/Smurf1ᵉ osteoblasts
Time after injection (h)
0 4 8 16 24 48 72 96
(DSS)6 + derivative
(DSS)6-derivative
(NAA)6-derivative
Chalcone derivative

Supplementary Figure 16. Tissue distribution, osteoblast accumulation and dose-response pattern of different chalcone derivative formulations. (a) Quantitative analysis of different chalcone derivative formulations by LC-MS/MS in bones, livers and kidneys from BMP-2^n/Smurf1^e subgroup of 15-month-old osteoporotic mice (OVX at 6 months old) with intravenous administration of chalcone derivative, $(NAA)_6$-derivative, $(DSS)_6$-derivative and $(DSS)_6$ + derivative, respectively. The amounts of chalcone derivative formulations in different tissues were normalized to injected dose. **(b)** Quantitative analysis of chalcone derivative formulations by LC-MS/MS in Alp^+, Alp^-, osteocalcin$^+$, osteocalcin$^-$ and Oscar$^+$ cells (sorted by FACS). The amounts of chalcone derivative formulations in different cells were normalized to mean value of Alp^+ or osteocalcin$^+$ cells from the mice with intravenous administration of the chalcone derivative. **(c)** Relative level of Smurf1 activity (Smad1 bound to Smurf1, ubiquitination of Smad1 and Runx2) in osteocalcin$^+$ cells (sorted by FACS) from BMP-2^n/Smurf1^e subgroup of 15-month-old osteoporotic mice administered with different chalcone derivative formulations at a series of doses (2.5, 5.0, 10.0, 15.0 and 20.0 μmol kg^{-1}). **(d)** Relative level of Smurf1 activity (Smad1 bound to Smurf1, ubiquitination of Smad1 and Runx2), p-Smad1, Runx2 activation and osteocalcin mRNA in osteocalcin$^+$ cells (sorted by FACS) from BMP-2^n/Smurf1^e subgroup of 15-month-old osteoporotic mice administered with different chalcone derivative formulations (10.0 μmol kg^{-1}) at a series of time points (4, 8, 16, 24, 48, 72 and 96 h). *$P < 0.0001$ for $(DSS)_6$-derivative versus $(NAA)_6$-derivative at 16, 24, 48 and 72 h. Relative levels of Smad1 bound to Smurf1, p-Smad1, Runx2 activation, ubiquitination of Smad1 and Runx2 and osteocalcin mRNA were normalized to the mean values of osteocalcin$^+$ cells from osteoporotic BMP-2^n/Smurf1^e mice treated with vehicle. $n = 9$ per group. Data are mean ± s.d. followed by one-way ANOVA with a *post-hoc* test.

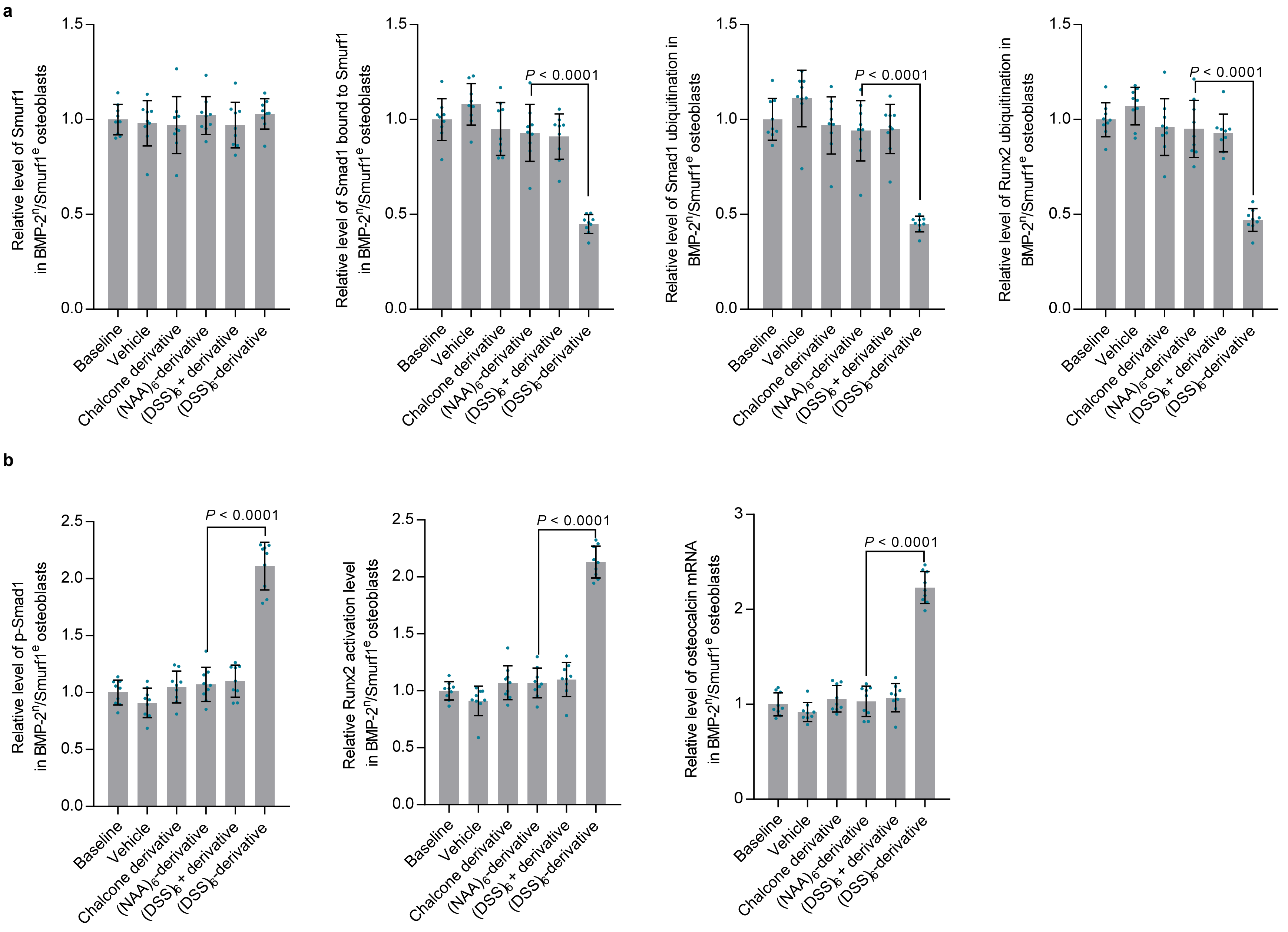

a
Relative level of Smurf1 in BMP-2n/Smurf1e osteoblasts
Relative level of Smad1 bound to Smurf1 in BMP-2n/Smurf1e osteoblasts
Relative level of Smad1 ubiquitination in BMP-2n/Smurf1e osteoblasts
Relative level of Runx2 ubiquitination in BMP-2n/Smurf1e osteoblasts
P < 0.0001
Baseline
Vehicle
Chalcone derivative
(NAA)6-derivative
(DSS)6 + derivative
(DSS)6-derivative
b
Relative level of p-Smad1 in BMP-2n/Smurf1e osteoblasts
Relative Runx2 activation level in BMP-2n/Smurf1e osteoblasts
Relative level of osteocalcin mRNA in BMP-2n/Smurf1e osteoblasts

Supplementary Figure 17. Effects of different chalcone derivative formulations on Smurf1 activity and BMP signaling in osteoblasts from mice BMP-2^{n}/Smurf1^{e} subgroup. (a) Relative levels of Smurf1 expression and Smurf1 activity (Smad1 bound to Smurf1, ubiquitination of Smad1 and Runx2) in osteoblasts isolated from BMP-2^{n}/Smurf1^{e} subgroup of 15-month-old osteoporotic mice (OVX at 6 months old) administrated with vehicle, chalcone derivative, $(NAA)_6$-chalcone derivative, $(DSS)_6$-chalcone derivative and $(DSS)_6$ + chalcone derivative, at a dose of 10.0 μmol kg^{-1}, respectively. **(b)** Relative levels of p-Smad1, Runx2 activation and osteocalcin mRNA in the above osteoblasts. Relative levels of Smurf1 and p-Smad1, Smad1 bound to Smurf1, Runx2 activation, ubiquitination of Smad1 and Runx2 and osteocalcin mRNA were normalized to the mean values of osteoblasts before the treatment (baseline). n = 9 per group. Data are mean ± s.d. followed by one-way ANOVA with a *post-hoc* test.

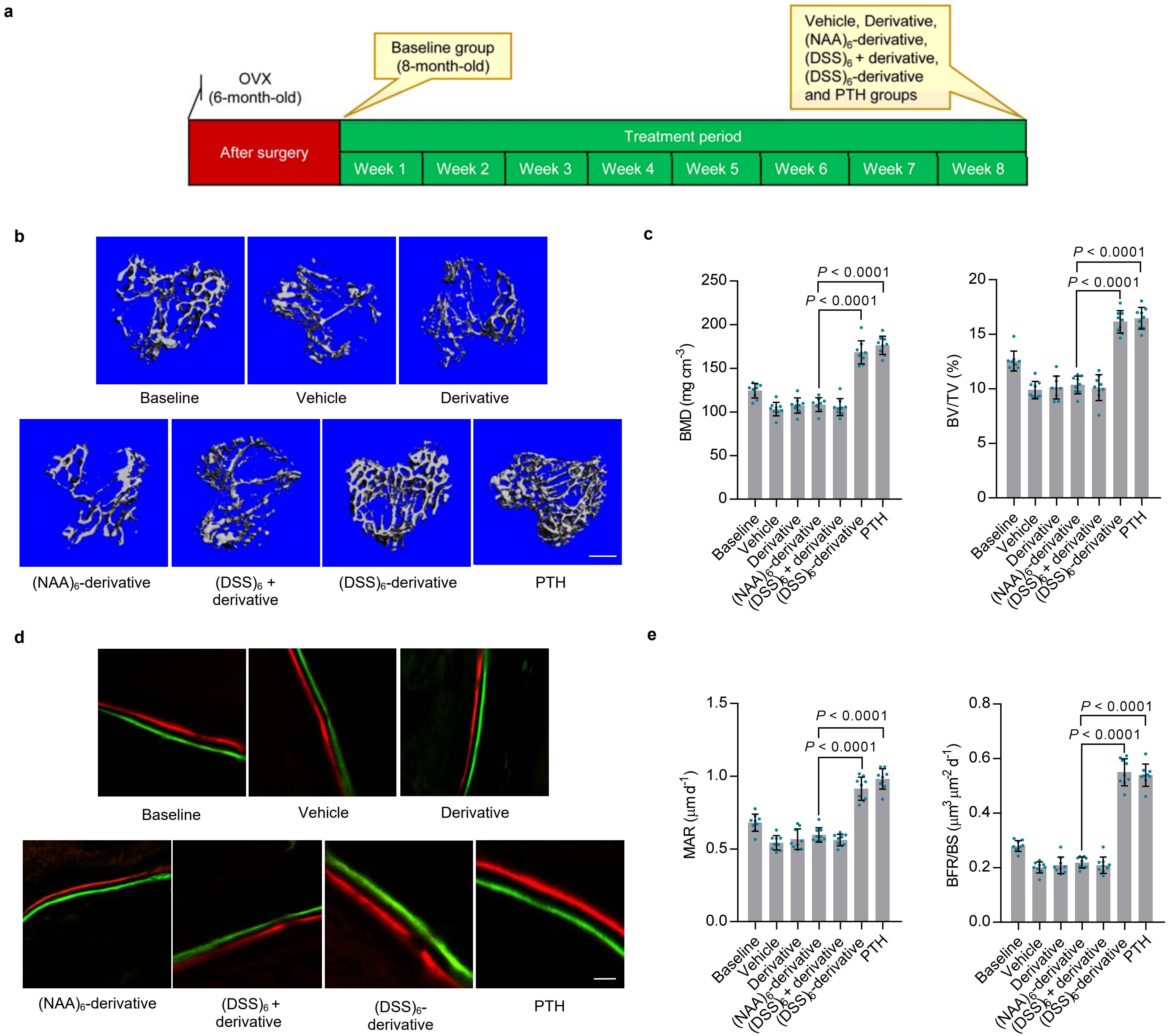
a
OVX
(6-month-old)
Baseline group
(8-month-old)
Vehicle, Derivative,
(NAA)6-derivative,
(DSS)6 + derivative,
(DSS)6-derivative
and PTH groups
After surgery
Treatment period
Week 1
Week 2
Week 3
Week 4
Week 5
Week 6
Week 7
Week 8
b
Baseline
Vehicle
Derivative
(NAA)6-derivative
(DSS)6 + derivative
(DSS)6-derivative
PTH
c
BMD (mg cm-3)
BV/TV (%)
P < 0.0001
d
Baseline
Vehicle
Derivative
(NAA)6-derivative
(DSS)6 + derivative
(DSS)6-derivative
PTH
e
MAR (µm d-1)
BFR/BS (µm3 µm2 d-1)
P < 0.0001

f

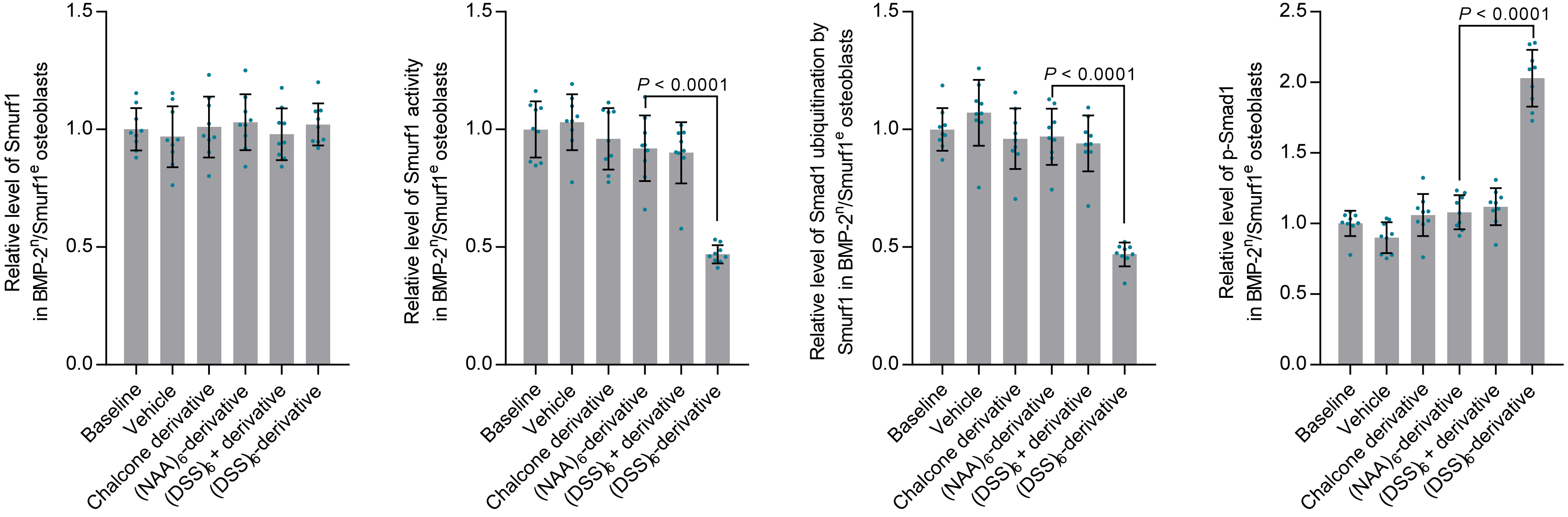
Relative level of Smurf1 in BMP-2^{n}/Smurf1^{e} osteoblasts
1.5
1.0
0.5
0.0
Relative level of Smurf1 activity in BMP-2^{n}/Smurf1^{e} osteoblasts
P < 0.0001
Relative level of Smad1 ubiquitination by Smurf1 in BMP-2^{n}/Smurf1^{e} osteoblasts
P < 0.0001
Relative level of p-Smad1 in BMP-2^{n}/Smurf1^{e} osteoblasts
2.5
2.0
1.5
1.0
0.5
0.0
P < 0.0001
Baseline
Vehicle
Chalcone derivative
(NAA)$_6$-derivative
(DSS)$_6$ + derivative
(DSS)$_6$-derivative

g

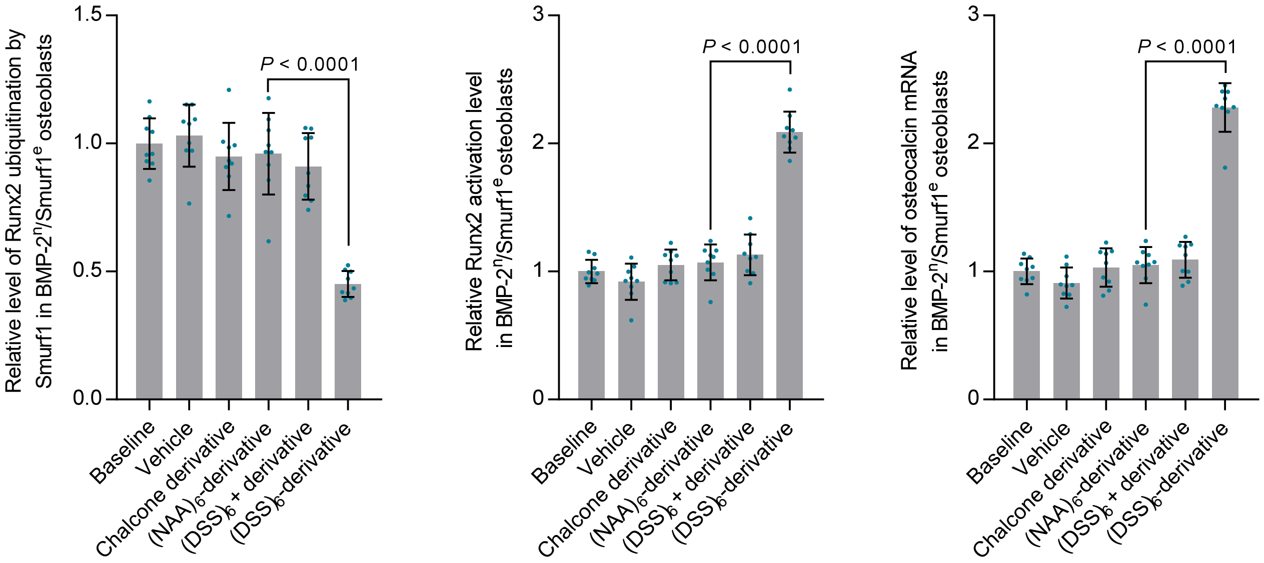
Relative level of Runx2 ubiquitination by Smurf1 in BMP-2^{n}/Smurf1^{e} osteoblasts
1.5
1.0
0.5
0.0
P < 0.0001
Relative Runx2 activation level in BMP-2^{n}/Smurf1^{e} osteoblasts
3
2
1
0
P < 0.0001
Relative level of osteocalcin mRNA in BMP-2^{n}/Smurf1^{e} osteoblasts
P < 0.0001
Baseline
Vehicle
Chalcone derivative
(NAA)$_6$-derivative
(DSS)$_6$ + derivative
(DSS)$_6$-derivative

Supplementary Figure 18. Effects of different chalcone derivative formulations on Smurf1 activity, BMP signaling and systemic bone formation in mice BMP-2^n/Smurf1^e subgroup. (a) A schematic diagram illustrating the experimental design. 6-month-old mice were OVX and left untreated for 2 months (8-month-old) to induce osteoporosis. The mice received intravenous administration of the chalcone derivative formulations at a dose of 10.0 μmol kg^{-1}, with an injection interval of once every three days, or daily subcutaneous injection of recombinant human parathyroid hormone (PTH, amino acids 1-34) at a dose of 40.0 μg kg^{-1}. **(b)** Representative images showing three-dimensional trabecular architecture by microCT reconstruction at the proximal tibia. Scale bars, 500 μm. **(c)** MicroCT measurements for BMD and BV/TV at the proximal tibia. **(d)** Representative images showing bone formation at the proximal tibia assessed by xylenol (red) and calcein (green) labeling. Scale bar, 10 μm. **(e)** Analysis of dynamic bone histomorphometric parameters (MAR and BFR/BS) at the proximal tibia. **(f)** Relative levels of Smurf1 expression and Smurf1 activity (Smad1 bound to Smurf1 and ubiquitination of Smad1 and Runx2) in osteoblasts isolated from BMP-2^n/Smurf1^e subgroup of osteoporotic mice administrated with vehicle, chalcone derivative, $(NAA)_6$-chalcone derivative, $(DSS)_6$-chalcone derivative and $(DSS)_6$ + chalcone derivative, respectively. **(g)** Relative levels of p-Smad1, Runx2 activation and osteocalcin mRNA in the above osteoblasts. Relative levels of Smurf1 and p-Smad1, Smad1 bound to Smurf1, Runx2 activation, ubiquitination of Smad1 and Runx2 and osteocalcin mRNA were normalized to the mean values of osteoblasts before the treatment (baseline). n = 9 per group. Data are mean ± s.d. followed by one-way ANOVA with a *post-hoc* test.

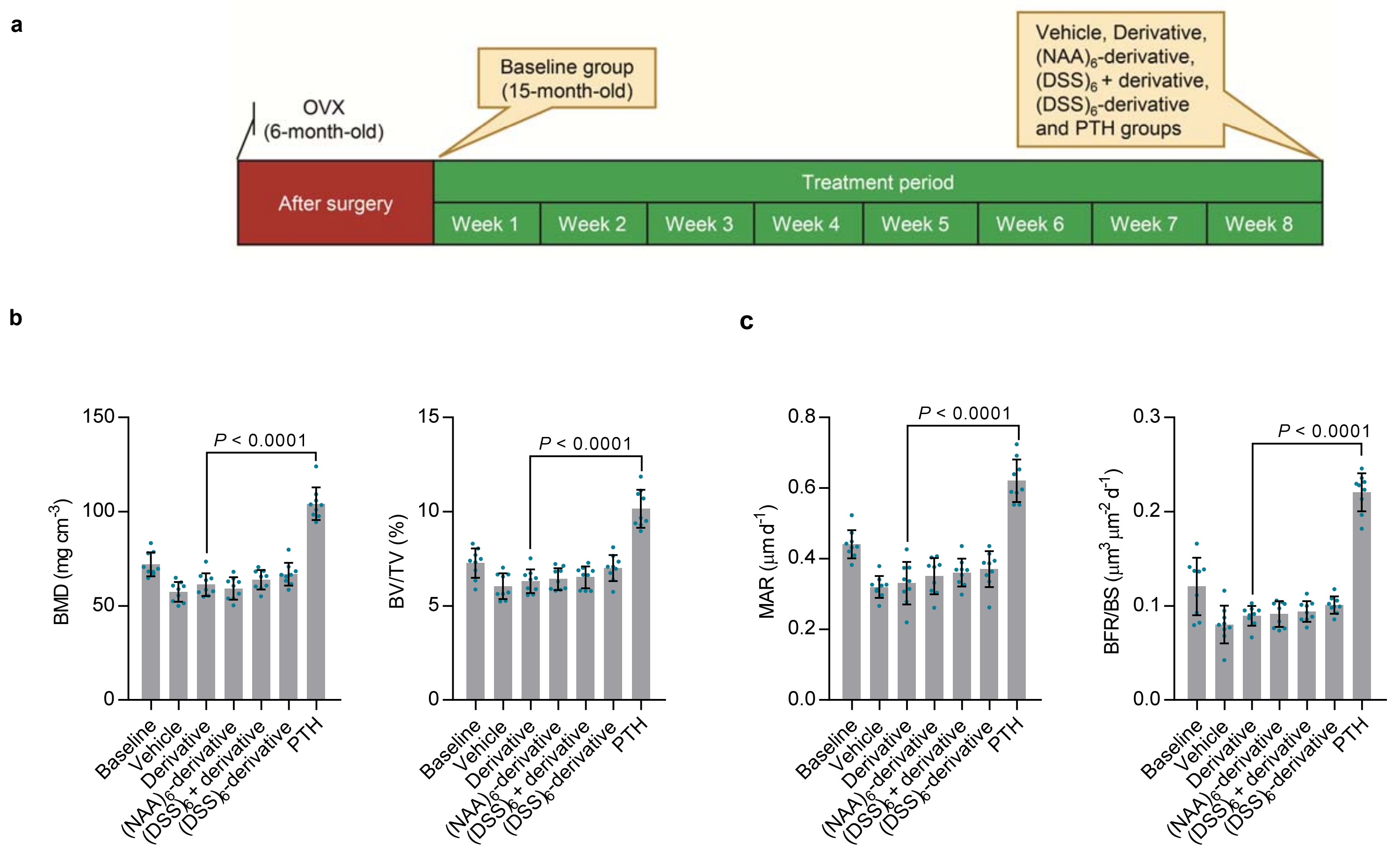

Supplementary Figure 19. Effects of different chalcone derivative formulations on systemic bone formation in mice BMP-2[d]/Smurf1[n] subgroup. (a) A schematic diagram illustrating the experimental design. 6-month-old mice were OVX and left untreated for 9 months (15-month-old) to induce osteoporosis. The mice received intravenous administration of the chalcone derivative formulations at a dose of 10.0 μmol kg^{-1}, with an injection interval of once every three days, or subcutaneous injection of recombinant human parathyroid hormone (PTH, amino acids 1-34) at a dose of 40.0 μg kg^{-1}. **(b)** microCT measurements for BMD and BV/TV at the proximal tibia. Scale bars, 500 μm. **(c)** Analysis of dynamic bone histomorphometric parameters (MAR and BFR/BS) at the proximal tibia. n = 9 per group. Data are mean ± s.d. followed by one-way ANOVA with a *post-hoc* test.

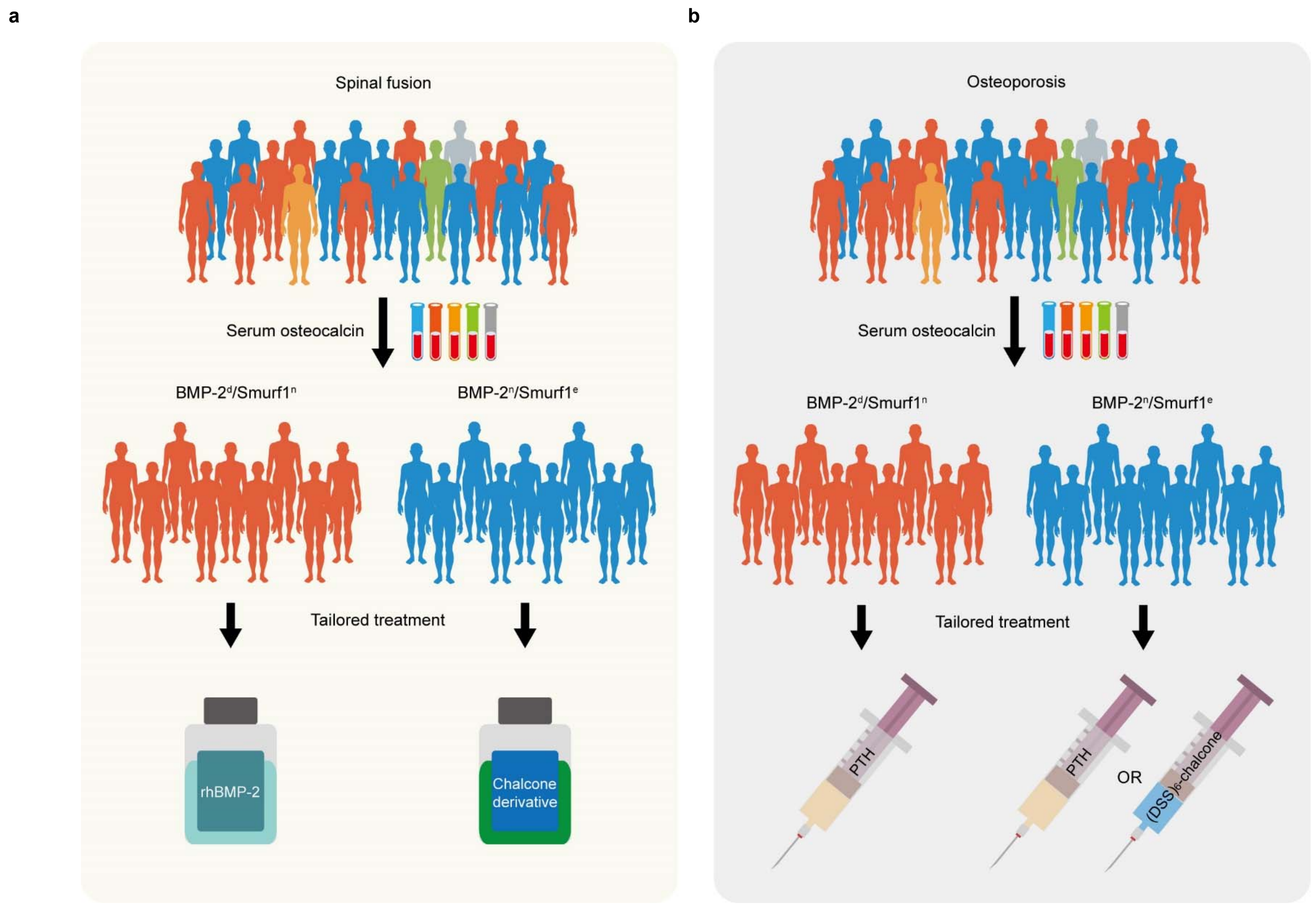

Supplementary Figure 20. Proposed precision medicine-based bone anabolic strategy. Age-related osteoporotic population could be classified into different subgroups by a serum osteocalcin-based stratification method. **(a)** Traditional rhBMP-2 could be continuously used to promote local bone formation in BMP-2^{d}/Smurf1^{n} subgroup during spinal fusion, while chalcone derivative is a promising tailored treatment for spinal fusion in BMP-2^{n}/Smurf1^{e} subgroup. **(b)** For systemic bone formation, $(DSS)_6$-chalcone derivative could be an alternative therapeutic in BMP-2^{n}/Smurf1^{e} subgroup besides the standard bone anabolic agent PTH in treatment of age-related osteoporosis.

Supplementary Fig. 8f

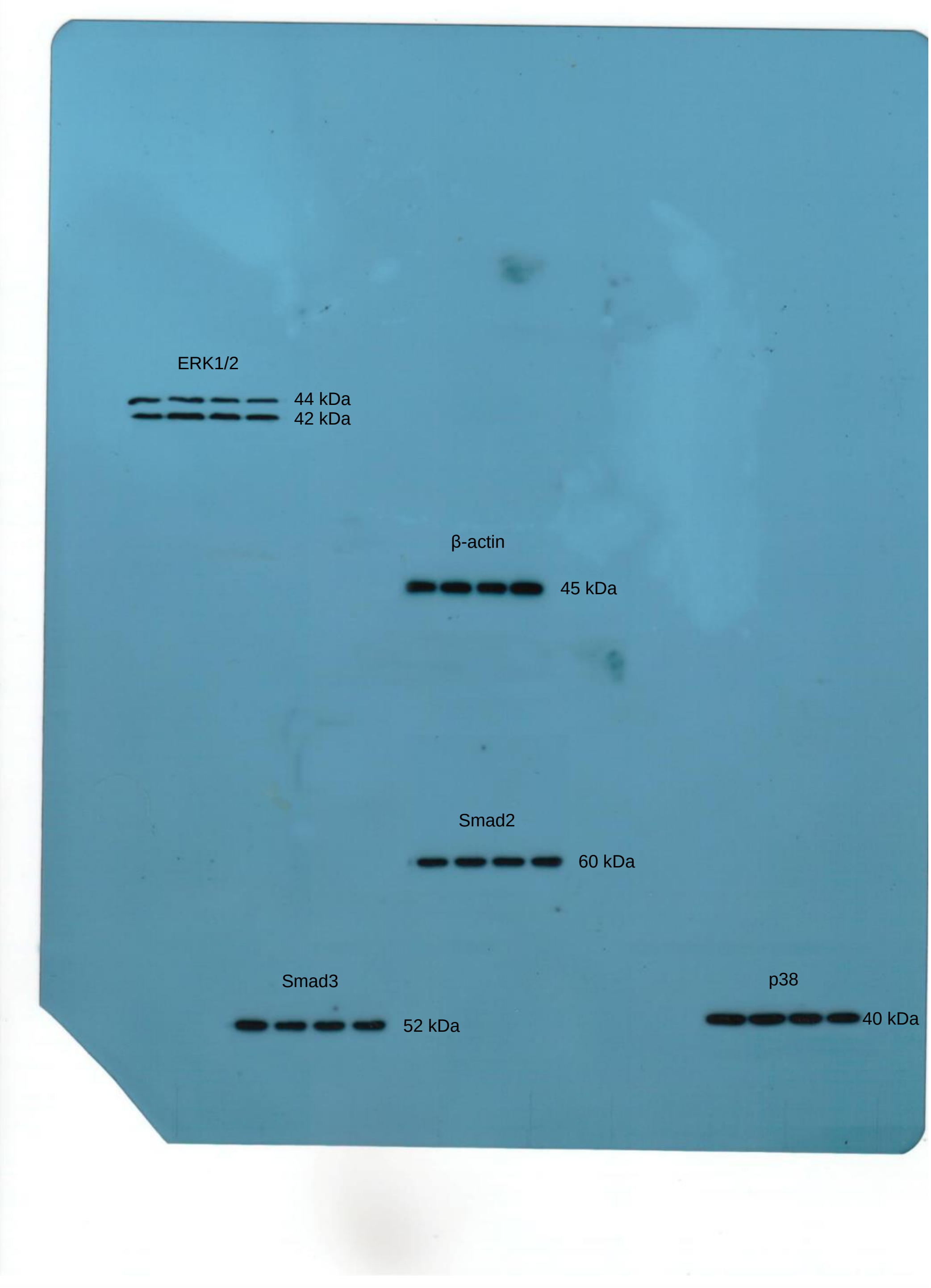

Supplementary Fig. 8f

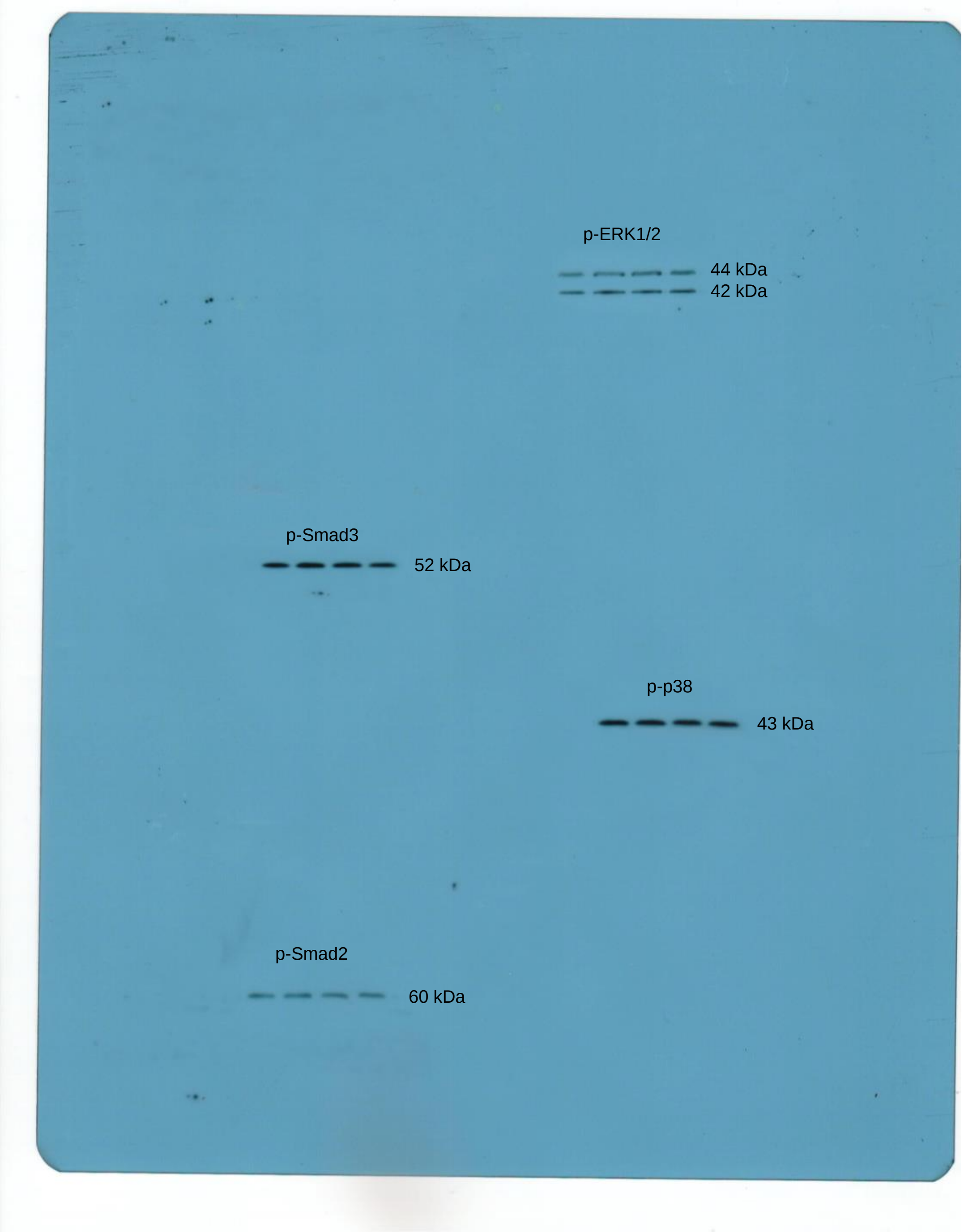

Supplementary Fig. 9c

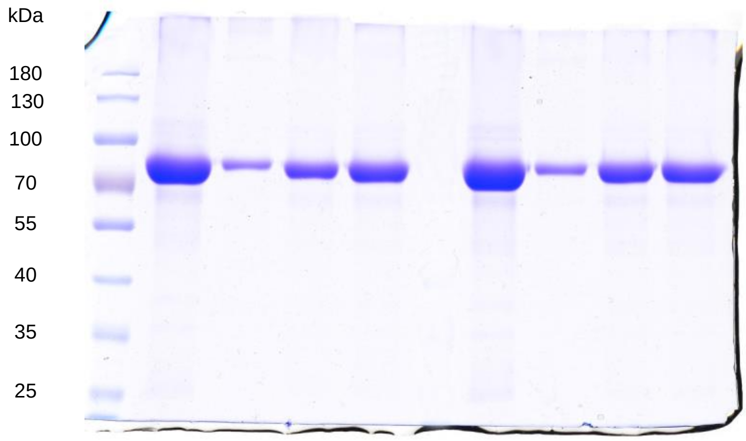

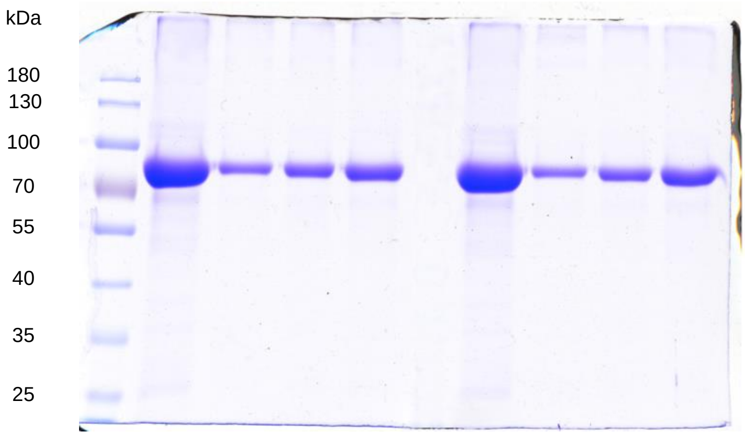

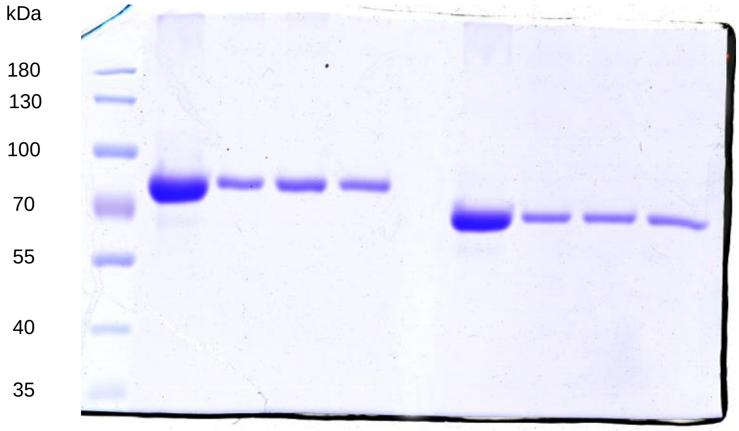

Supplementary Fig. 21. The uncropped western blots and gels.

Supplementary Table 1. Clinical features of aged osteoporotic VCF and adult traumatic VCF patients

Patient No.	Age	Gender	T score for BMD at lumbar spine	Diagnosis	Surgical operation
1	67	Female	-2.3	L1 fragile fracture	Percutaneous vertebroplasty
2	61	Female	-2.4	L2 fragile fracture	Fracture reduction and internal fixation
3	63	Male	-2.6	L4 fragile fracture	Percutaneous kyphoplasty
4	65	Female	-3.1	T12 fragile fracture	Percutaneous kyphoplasty
5	61	Female	-2.2	T12 fragile fracture	Fracture reduction and internal fixation
6	62	Female	-2.1	L4 fragile fracture	Fracture reduction and internal fixation
7	64	Male	-2.6	T10/T11 fragile fracture	Percutaneous kyphoplasty
8	62	Female	-2.3	L2/L4 fragile fracture	Fracture reduction and internal fixation
9	63	Male	-2.4	L1 fragile fracture	Percutaneous vertebroplasty
10	68	Male	-2.5	L2 fragile fracture	Percutaneous kyphoplasty
11	61	Male	-2.3	L3 fragile fracture	Percutaneous kyphoplasty
12	62	Female	-1.9	L5 fragile fracture	Percutaneous vertebroplasty
13	68	Female	-3.0	L1/L2 fragile fracture	Percutaneous kyphoplasty
14	64	Male	-2.7	L4 fragile fracture	Percutaneous kyphoplasty
15	63	Female	-2.0	T10 fragile fracture	Fracture reduction and internal fixation
16	68	Female	-2.5	T11/L2 fragile fracture	Percutaneous vertebroplasty
17	69	Male	-2.3	L1 fragile fracture	Percutaneous vertebroplasty
18	63	Female	-2.6	L3 fragile fracture	Fracture reduction and internal fixation
19	61	Female	-2.2	L2/L4 fragile fracture	Percutaneous kyphoplasty
20	64	Female	-2.5	T8 fragile fracture	Percutaneous kyphoplasty
21	62	Male	-2.3	T12 fragile fracture	Percutaneous vertebroplasty
22	69	Male	-2.8	L1/L4 fragile fracture	Percutaneous kyphoplasty
23	62	Female	-1.9	L4 fragile fracture	Percutaneous vertebroplasty
24	64	Female	-2.5	L1 fragile fracture	Percutaneous vertebroplasty

25	61	Female	-2.0	L2/L3 fragile fracture	Percutaneous vertebroplasty
26	69	Male	-2.6	L2/L5 fragile fracture	Percutaneous kyphoplasty
27	65	Male	-2.4	T9 fragile fracture	Percutaneous kyphoplasty
28	62	Female	-2.3	T11 fragile fracture	Fracture reduction and internal fixation
29	64	Male	-2.6	L3 fragile fracture	Percutaneous kyphoplasty
30	60	Female	-2.5	L2/L4 fragile fracture	Percutaneous kyphoplasty
31	62	Female	-2.4	T12/L3 fragile fracture	Percutaneous kyphoplasty
32	66	Male	-2.9	L1 fragile fracture	Percutaneous kyphoplasty
33	64	Female	-2.5	L4 fragile fracture	Percutaneous kyphoplasty
34	69	Female	-2.6	T11 fragile fracture	Percutaneous vertebroplasty
35	61	Female	-2.1	L2 fragile fracture	Percutaneous vertebroplasty
36	64	Male	-2.3	T12 fragile fracture	Percutaneous vertebroplasty
37	68	Female	-2.5	T12 fragile fracture	Percutaneous kyphoplasty
38	63	Female	-2.7	L2 fragile fracture	Fracture reduction and internal fixation
39	60	Male	-2.8	L1 fragile fracture	Percutaneous kyphoplasty
40	61	Female	-2.2	T8 fragile fracture	Percutaneous vertebroplasty
41	64	Male	-2.4	T10 fragile fracture	Percutaneous kyphoplasty
42	65	Female	-2.6	T12/L4 fragile fracture	Percutaneous vertebroplasty
43	67	Female	-2.4	L1 fragile fracture	Percutaneous kyphoplasty
44	66	Female	-2.4	T11 fragile fracture	Percutaneous kyphoplasty
45	62	Female	-2.5	T9 fragile fracture	Percutaneous kyphoplasty
46	61	Female	-1.9	L2/L3 fragile fracture	Percutaneous vertebroplasty
47	69	Male	-2.5	L1 fragile fracture	Percutaneous kyphoplasty
48	66	Male	-2.6	L4 fragile fracture	Percutaneous kyphoplasty
49	65	Female	-2.1	T12 fragile fracture	Percutaneous vertebroplasty
50	67	Female	-2.4	T12/L3 fragile fracture	Percutaneous kyphoplasty

51	68	Female	-2.9	L5 fragile fracture	Percutaneous vertebroplasty
52	61	Female	-2.7	L4 fragile fracture	Percutaneous vertebroplasty
53	63	Female	-1.9	L1 fragile fracture	Fracture reduction and internal fixation
54	68	Male	-2.4	T10 fragile fracture	Percutaneous kyphoplasty
55	64	Female	-2.5	T11 fragile fracture	Percutaneous kyphoplasty
56	64	Female	-2.8	L1 fragile fracture	Percutaneous kyphoplasty
57	67	Male	-2.2	L2 fragile fracture	Percutaneous vertebroplasty
58	66	Female	-2.6	L1 fragile fracture	Percutaneous kyphoplasty
59	66	Male	-1.8	L2 fragile fracture	Percutaneous vertebroplasty
60	65	Male	-2.7	L2 fragile fracture	Percutaneous kyphoplasty
61	68	Female	-2.5	L2 fragile fracture	Percutaneous vertebroplasty
62	67	Female	-2.2	L5 fragile fracture	Percutaneous kyphoplasty
63	65	Female	-2.1	L1 fragile fracture	Percutaneous kyphoplasty
64	66	Female	-3.0	L2 fragile fracture	Fracture reduction and internal fixation
65	64	Male	-2.6	T11 fragile fracture	Percutaneous kyphoplasty
66	68	Female	-3.6	T12 fragile fracture	Percutaneous kyphoplasty
67	67	Male	-3.2	T10/L2 fragile fracture	Percutaneous kyphoplasty
68	66	Female	-2.5	T11 fragile fracture	Percutaneous kyphoplasty
69	65	Male	-2.6	L5 fragile fracture	Percutaneous vertebroplasty
70	67	Female	-2.8	L2 fragile fracture	Percutaneous kyphoplasty
71	67	Female	-2.0	L1/L4 fragile fracture	Percutaneous kyphoplasty
72	63	Female	-1.9	L2 fragile fracture	Fracture reduction and internal fixation
73	68	Female	-2.6	L1 fragile fracture	Percutaneous kyphoplasty
74	67	Male	-2.4	T12 fragile fracture	Percutaneous kyphoplasty
75	63	Male	-1.9	L1 fragile fracture	Percutaneous kyphoplasty
76	71	Female	-2.5	L2 fragile fracture	Percutaneous kyphoplasty

77	72	Female	-2.0	L4 fragile fracture	Percutaneous kyphoplasty
78	70	Female	-2.4	L1 fragile fracture	Percutaneous kyphoplasty
79	76	Male	-3.6	L5 fragile fracture	Percutaneous kyphoplasty
80	70	Male	-2.1	T11 fragile fracture	Percutaneous kyphoplasty
81	73	Female	-3.2	T12 fragile fracture	Percutaneous vertebroplasty
82	74	Female	-2.4	L1 fragile fracture	Percutaneous kyphoplasty
83	71	Male	-2.2	L1/L4 fragile fracture	Percutaneous kyphoplasty
84	70	Female	-2.0	L2 fragile fracture	Percutaneous vertebroplasty
85	75	Female	-2.8	T11/L2 fragile fracture	Percutaneous kyphoplasty
86	78	Male	-3.3	L3 fragile fracture	Percutaneous kyphoplasty
87	76	Male	-3.4	L2 fragile fracture	Percutaneous kyphoplasty
88	74	Female	-2.3	L5 fragile fracture	Percutaneous vertebroplasty
89	73	Female	-2.5	T10/L4 fragile fracture	Percutaneous kyphoplasty
90	72	Female	-2.6	L1 fragile fracture	Percutaneous kyphoplasty
91	70	Male	-2.1	L1 fragile fracture	Percutaneous vertebroplasty
92	71	Male	-2.4	L1 fragile fracture	Percutaneous kyphoplasty
94	72	Female	-3.1	L4 fragile fracture	Percutaneous vertebroplasty
94	73	Male	-2.8	L5 fragile fracture	Percutaneous kyphoplasty
95	75	Male	-3.2	T7 fragile fracture	Percutaneous kyphoplasty
96	79	Female	-4.1	T12 fragile fracture	Percutaneous kyphoplasty
97	78	Female	-3.4	L1 fragile fracture	Percutaneous kyphoplasty
98	77	Male	-2.8	L1 fragile fracture	Percutaneous kyphoplasty
99	72	Female	-2.4	L4 fragile fracture	Percutaneous vertebroplasty
100	77	Male	-3.6	T10 fragile fracture	Percutaneous kyphoplasty
101	78	Female	-3.8	T12 fragile fracture	Percutaneous kyphoplasty
102	71	Female	-2.4	L2 fragile fracture	Percutaneous vertebroplasty

103	70	Male	-2.5	L3 fragile fracture	Percutaneous kyphoplasty
104	74	Female	-3.1	L3 fragile fracture	Percutaneous kyphoplasty
105	73	Female	-2.6	L4 fragile fracture	Percutaneous kyphoplasty
106	75	Male	-2.5	T11/L2 fragile fracture	Percutaneous kyphoplasty
107	76	Male	-2.6	T9 fragile fracture	Percutaneous kyphoplasty
108	75	Female	-3.2	L1 fragile fracture	Percutaneous kyphoplasty
109	71	Female	-2.5	L3 fragile fracture	Percutaneous kyphoplasty
110	77	Male	-3.1	L2 fragile fracture	Percutaneous kyphoplasty
111	78	Male	-2.6	L4 fragile fracture	Percutaneous kyphoplasty
112	75	Male	-3.8	L4 fragile fracture	Percutaneous kyphoplasty
113	79	Female	-3.4	T10/L4 fragile fracture	Percutaneous kyphoplasty
114	73	Female	-2.4	L1 fragile fracture	Percutaneous vertebroplasty
115	71	Male	-2.5	L2 fragile fracture	Percutaneous kyphoplasty
116	70	Female	-1.9	L3 fragile fracture	Percutaneous vertebroplasty
117	70	Female	-2.5	L3 fragile fracture	Percutaneous kyphoplasty
118	78	Male	-2.7	T11/L3 fragile fracture	Percutaneous kyphoplasty
119	76	Female	-3.2	L2 fragile fracture	Percutaneous kyphoplasty
120	78	Female	-2.7	L3 fragile fracture	Percutaneous kyphoplasty
121	71	Male	-2.5	L2 fragile fracture	Percutaneous kyphoplasty
122	77	Female	-2.5	L4 fragile fracture	Percutaneous kyphoplasty
123	76	Female	-2.8	L4 fragile fracture	Percutaneous kyphoplasty
124	78	Female	-2.5	L1/L3 fragile fracture	Percutaneous kyphoplasty
125	77	Male	-3.1	L2 fragile fracture	Percutaneous kyphoplasty
126	74	Female	-2.7	L2/L5 fragile fracture	Percutaneous kyphoplasty
127	73	Female	-2.6	L1 fragile fracture	Percutaneous kyphoplasty
128	72	Female	-2.2	T12 fragile fracture	Percutaneous kyphoplasty

129	72	Female	-2.1	T11/L3 fragile fracture	Percutaneous vertebroplasty
130	71	Male	-2.6	T12 fragile fracture	Percutaneous kyphoplasty
131	73	Female	-2.5	T11 fragile fracture	Percutaneous kyphoplasty
132	73	Female	-2.3	T12 fragile fracture	Percutaneous kyphoplasty
133	78	Male	-2.4	L1 fragile fracture	Percutaneous kyphoplasty
134	79	Female	-4.2	L2 fragile fracture	Percutaneous kyphoplasty
135	76	Male	-2.5	L3 fragile fracture	Percutaneous kyphoplasty
136	75	Male	-2.2	L2/L4 fragile fracture	Percutaneous vertebroplasty
137	78	Female	-2.1	L2/L5 fragile fracture	Percutaneous vertebroplasty
138	77	Male	-3.5	T8 fragile fracture	Percutaneous kyphoplasty
139	75	Female	-2.5	T10 fragile fracture	Percutaneous kyphoplasty
140	74	Female	-2.7	L1 fragile fracture	Percutaneous kyphoplasty
141	76	Female	-2.8	L2/L4 fragile fracture	Percutaneous kyphoplasty
142	79	Male	-3.4	L3 fragile fracture	Percutaneous kyphoplasty
143	78	Male	-2.5	L2 fragile fracture	Percutaneous kyphoplasty
144	74	Female	-2.4	L2 fragile fracture	Percutaneous kyphoplasty
145	73	Female	-2.6	L1 fragile fracture	Percutaneous kyphoplasty
146	72	Male	-2.4	T12 fragile fracture	Percutaneous kyphoplasty
147	71	Female	-2.2	T12/L2 fragile fracture	Percutaneous vertebroplasty
148	78	Male	-2.4	T11 fragile fracture	Percutaneous vertebroplasty
149	79	Male	-2.8	L2 fragile fracture	Percutaneous kyphoplasty
150	75	Female	-2.4	L2 fragile fracture	Percutaneous kyphoplasty
151	31	Male	-0.4	Traumatic VCF (L1)	Internal fixation
152	30	Male	0.8	Traumatic VCF (T12)	Internal fixation
153	30	Male	0.4	Traumatic VCF (L2)	Conservative treatment
154	31	Male	-0.2	Traumatic VCF (L2)	Internal fixation

155	35	Female	0.7	Traumatic VCF (T12)	Internal fixation
156	37	Male	-0.5	Traumatic VCF (T12)	Internal fixation
157	32	Female	0.7	Traumatic VCF (L2)	Internal fixation
158	34	Female	0.2	Traumatic VCF (L3)	Internal fixation
159	30	Male	1.0	Traumatic VCF (T11/L2)	Internal fixation
160	36	Male	-0.4	Traumatic VCF (L1)	Internal fixation
161	34	Male	0.6	Traumatic VCF (L1)	Conservative treatment
162	33	Female	0.7	Traumatic VCF (L2)	Internal fixation
163	38	Female	-1.0	Traumatic VCF (T12)	Internal fixation
164	33	Male	-0.4	Traumatic VCF (T12/L2)	Internal fixation
165	33	Female	-0.2	Traumatic VCF (L2)	Internal fixation
166	31	Female	0.6	Traumatic VCF (L3)	Conservative treatment
167	32	Female	-0.2	Traumatic VCF (L1)	Internal fixation
168	36	Male	0.6	Traumatic VCF (L2)	Internal fixation
169	33	Male	-0.1	Traumatic VCF (T11/L3)	Internal fixation
170	39	Male	-0.8	Traumatic VCF (L1)	Internal fixation
171	35	Female	-0.5	Traumatic VCF (L2)	Internal fixation
172	31	Male	1.0	Traumatic VCF (L3)	Conservative treatment
173	39	Male	0.2	Traumatic VCF (L1)	Internal fixation
174	37	Male	-0.3	Traumatic VCF (L1/L2)	Internal fixation
175	37	Female	-0.5	Traumatic VCF (L2)	Internal fixation
176	39	Female	-1.4	Traumatic VCF (L3)	Internal fixation
177	30	Male	0.6	Traumatic VCF (L2)	Conservative treatment
178	33	Male	-0.2	Traumatic VCF (L2)	Conservative treatment
179	35	Female	0.6	Traumatic VCF (L1)	Internal fixation
180	36	Male	-0.2	Traumatic VCF (L2)	Internal fixation

181	38	Male	-0.7	Traumatic VCF (L2)	Internal fixation
182	38	Male	0.2	Traumatic VCF (L1)	Internal fixation
183	39	Female	-1.2	Traumatic VCF (L4)	Internal fixation
184	33	Female	0.6	Traumatic VCF (L1)	Conservative treatment
185	30	Male	1.5	Traumatic VCF (T12)	Conservative treatment
186	36	Male	-0.3	Traumatic VCF (L3)	Internal fixation
187	34	Male	0.5	Traumatic VCF (T11/L2)	Internal fixation
188	31	Female	-0.9	Traumatic VCF (L1)	Internal fixation
189	39	Male	0.2	Traumatic VCF (L2)	Internal fixation
190	38	Male	-0.3	Traumatic VCF (L1)	Internal fixation
191	32	Female	0.8	Traumatic VCF (L2)	Conservative treatment

Supplementary Table 2. Clinical features of aged osteoporotic LSS and adult LDH patients

Patient No.	Age	Gender	T score for BMD at lumbar spine	Diagnosis	Surgical operation
1	62	Female	-2.3	Lumbar spinal stenosis (L4/5)	Laminectomy and spinal fusion
2	67	Female	-2.5	Lumbar spinal stenosis and disc herniation (L4/5)	Laminectomy and spinal fusion
3	68	Male	-2.6	Lumbar spinal stenosis (L5/S1)	Laminectomy and spinal fusion
4	64	Female	-2.3	Lumbar spinal stenosis (L4/5)	Laminectomy and spinal fusion
5	66	Female	-2.5	Lumbar spinal stenosis and disc herniation (L4/5)	Laminectomy and spinal fusion
6	65	Male	-2.2	Lumbar spinal stenosis and disc herniation (L4/5 and L5/S1)	Laminectomy and spinal fusion
7	66	Female	-2.5	Lumbar spinal stenosis (L5/S1)	Laminectomy and spinal fusion
8	68	Male	-2.8	Lumbar spinal stenosis (L5/S1)	Laminectomy and spinal fusion
9	60	Female	-2.5	Lumbar spinal stenosis (L4/5)	Laminectomy and spinal fusion
10	68	Male	-2.5	Lumbar spinal stenosis (L3/4 and L4/5)	Laminectomy and spinal fusion
11	69	Female	-3.4	Lumbar spinal stenosis (L4/5 and L5/S1)	Laminectomy and spinal fusion
12	68	Male	-2.3	Lumbar spinal stenosis and disc herniation (L5/S1)	Laminectomy and spinal fusion
13	64	Female	-2.5	Lumbar spinal stenosis and disc herniation (L4/5)	Laminectomy and spinal fusion
14	63	Female	-2.7	Lumbar spinal stenosis (L4/5 and L5/S1)	Laminectomy and spinal fusion
15	61	Female	-2.3	Lumbar spinal stenosis and disc herniation (L4/5 and L5/S1)	Laminectomy and spinal fusion
16	66	Male	-2.0	Lumbar spinal stenosis (L4/5)	Laminectomy and spinal fusion
17	68	Female	-2.4	Lumbar spinal stenosis (L4/5)	Laminectomy and spinal fusion
18	67	Female	-1.9	Lumbar spinal stenosis (L4/5)	Laminectomy and spinal fusion
19	60	Male	-2.4	Lumbar spinal stenosis (L4/5 and L5/S1)	Laminectomy and spinal fusion
20	63	Female	-2.5	Lumbar spinal stenosis and disc herniation (L3/4 and L4/5)	Laminectomy and spinal fusion
21	69	Female	-2.3	Lumbar spinal stenosis (L4/5)	Laminectomy and spinal fusion
22	68	Female	-2.5	Lumbar spinal stenosis (L5/S1)	Laminectomy and spinal fusion
23	62	Female	-2.6	Lumbar spinal stenosis (L4/5 and L5/S1)	Laminectomy and spinal fusion

24	63	Male	-2.1	Lumbar spinal stenosis (L4/5)	Laminectomy and spinal fusion
25	63	Male	-1.8	Lumbar spinal stenosis and disc herniation (L5/S1)	Laminectomy and spinal fusion
26	65	Female	-2.4	Lumbar spinal stenosis and disc herniation (L4/5 and L5/S1)	Laminectomy and spinal fusion
27	65	Female	-2.7	Lumbar spinal stenosis (L4/5)	Laminectomy and spinal fusion
28	68	Male	-2.5	Lumbar spinal stenosis and disc herniation (L4/5 and L5/S1)	Laminectomy and spinal fusion
29	69	Male	-3.1	Lumbar spinal stenosis and disc herniation (L4/5)	Laminectomy and spinal fusion
30	61	Female	-2.6	Lumbar spinal stenosis (L4/5)	Laminectomy and spinal fusion
31	67	Male	-2.3	Lumbar spinal stenosis and disc herniation (L3/4 and L4/5)	Laminectomy and spinal fusion
32	62	Female	-2.2	Lumbar spinal stenosis and disc herniation (L4/5)	Laminectomy and spinal fusion
33	62	Female	-2.2	Lumbar spinal stenosis (L3/4 and L4/5)	Laminectomy and spinal fusion
34	64	Female	-2.4	Lumbar spinal stenosis (L5/S1)	Laminectomy and spinal fusion
35	65	Female	-2.5	Lumbar spinal stenosis (L5/S1)	Laminectomy and spinal fusion
36	66	Male	-2.9	Lumbar spinal stenosis and disc herniation (L4/5)	Laminectomy and spinal fusion
37	61	Male	-2.3	Lumbar spinal stenosis and disc herniation (L4/5)	Laminectomy and spinal fusion
38	68	Female	-2.5	Lumbar spinal stenosis and disc herniation (L4/5 and L5/S1)	Laminectomy and spinal fusion
39	69	Male	-2.3	Lumbar spinal stenosis (L5/S1)	Laminectomy and spinal fusion
40	62	Female	-2.5	Lumbar spinal stenosis (L4/5)	Laminectomy and spinal fusion
41	64	Male	-1.8	Lumbar spinal stenosis and disc herniation (L5/S1)	Laminectomy and spinal fusion
42	65	Female	-2.5	Lumbar spinal stenosis and disc herniation (L4/5)	Laminectomy and spinal fusion
43	65	Female	-2.4	Lumbar spinal stenosis (L5/S1)	Laminectomy and spinal fusion
44	67	Male	-3.3	Lumbar spinal stenosis (L4/5)	Laminectomy and spinal fusion
45	68	Female	-2.8	Lumbar spinal stenosis (L4/5)	Laminectomy and spinal fusion
46	68	Female	-2.5	Lumbar spinal stenosis and disc herniation (L4/5 and L5/S1)	Laminectomy and spinal fusion
47	69	Male	-2.7	Lumbar spinal stenosis (L4/5)	Laminectomy and spinal fusion
48	69	Male	-2.2	Lumbar spinal stenosis (L4/5)	Laminectomy and spinal fusion
49	65	Female	-2.5	Lumbar spinal stenosis (L3/4 and L4/5)	Laminectomy and spinal fusion

50	65	Male	-1.8	Lumbar spinal stenosis (L4/5)	Laminectomy and spinal fusion
51	64	Male	-2.0	Lumbar spinal stenosis and disc herniation (L 3/4 and L4/5)	Laminectomy and spinal fusion
52	64	Female	-2.5	Lumbar spinal stenosis and disc herniation (L4/5 and L5/S1)	Laminectomy and spinal fusion
53	65	Female	-2.5	Lumbar spinal stenosis (L4/5)	Laminectomy and spinal fusion
54	68	Female	-2.5	Lumbar spinal stenosis (L4/5)	Laminectomy and spinal fusion
55	66	Male	-2.2	Lumbar spinal stenosis (L4/5 and L5/S1)	Laminectomy and spinal fusion
56	65	Female	-2.5	Lumbar spinal stenosis (L4/5)	Laminectomy and spinal fusion
57	63	Male	-2.8	Lumbar spinal stenosis and disc herniation (L4/5 and L5/S1)	Laminectomy and spinal fusion
58	69	Female	-2.3	Lumbar spinal stenosis (L4/5)	Laminectomy and spinal fusion
59	63	Female	-2.5	Lumbar spinal stenosis and disc herniation (L4/5 and L5/S1)	Laminectomy and spinal fusion
60	62	Female	-1.9	Lumbar spinal stenosis (L5/S1)	Laminectomy and spinal fusion
61	68	Male	-2.7	Lumbar spinal stenosis (L4/5)	Laminectomy and spinal fusion
62	65	Female	-2.4	Lumbar spinal stenosis (L5/S1)	Laminectomy and spinal fusion
63	62	Female	-2.5	Lumbar spinal stenosis (L4/5)	Laminectomy and spinal fusion
64	68	Female	-2.3	Lumbar spinal stenosis (L4/5)	Laminectomy and spinal fusion
65	67	Male	-2.6	Lumbar spinal stenosis (L5/S1)	Laminectomy and spinal fusion
66	67	Male	-2.5	Lumbar spinal stenosis and disc herniation (L4/5 and L5/S1)	Laminectomy and spinal fusion
67	66	Female	-2.4	Lumbar spinal stenosis (L4/5)	Laminectomy and spinal fusion
68	69	Female	-2.6	Lumbar spinal stenosis (L4/5)	Laminectomy and spinal fusion
69	64	Female	-2.8	Lumbar spinal stenosis (L4/5 and L5/S1)	Laminectomy and spinal fusion
70	67	Male	-3.0	Lumbar spinal stenosis (L5/S1)	Laminectomy and spinal fusion
71	63	Male	-2.4	Lumbar spinal stenosis (L4/5)	Laminectomy and spinal fusion
72	68	Female	-2.2	Lumbar spinal stenosis (L5/S1)	Laminectomy and spinal fusion
73	69	Male	-2.7	Lumbar spinal stenosis and disc herniation (L4/5)	Laminectomy and spinal fusion
74	66	Female	-2.5	Lumbar spinal stenosis (L4/5)	Laminectomy and spinal fusion
75	37	Male	-0.4	Disc herniation (L4/5)	Disctomy and spinal fusion

76	38	Male	-0.2	Lumbar spinal stenosis and disc herniation (L4/5)	Laminectomy and spinal fusion
77	35	Female	0.4	L5/S1 disc herniation	Disctomy and spinal fusion
78	39	Male	-0.3	Lumbar spinal stenosis and disc herniation (L4/5)	Laminectomy and spinal fusion
79	39	Female	-0.6	Lumbar spinal stenosis and disc herniation (L5/S1)	Laminectomy and spinal fusion
80	32	Male	1.2	L4/5 disc herniation	Disctomy and spinal fusion
81	30	Male	1.4	L5/S1 disc herniation	Disctomy and spinal fusion
82	33	Female	-0.4	L4/5 disc herniation	Disctomy and spinal fusion
83	35	Female	-0.8	L4/5 disc herniation	Disctomy and spinal fusion
84	39	Female	-1.2	Lumbar spinal stenosis and disc herniation (L4/5)	Laminectomy and spinal fusion
85	31	Male	0.8	L5/S1 disc herniation	Disctomy and spinal fusion
86	31	Female	0.4	L4/5 disc herniation	Disctomy and spinal fusion
87	34	Male	0.5	L4/5 disc herniation	Disctomy and spinal fusion
88	39	Male	-0.7	Lumbar spinal stenosis and disc herniation (L4/5)	Laminectomy and spinal fusion
89	38	Female	-0.4	Lumbar spinal stenosis and disc herniation (L4/5)	Laminectomy and spinal fusion
90	32	Male	0.1	L5/S1 disc herniation	Disctomy and spinal fusion
91	33	Female	0.3	L4/5 disc herniation	Disctomy and spinal fusion
92	32	Female	0.8	L5/S1 disc herniation	Disctomy and spinal fusion
93	38	Male	-0.4	L4/5 disc herniation	Disctomy and spinal fusion
94	37	Male	0.6	L4/5 disc herniation	Disctomy and spinal fusion
95	39	Male	-0.9	Lumbar spinal stenosis and disc herniation (L4/5)	Laminectomy and spinal fusion
96	33	Male	1.2	L4/5 disc herniation	Disctomy and spinal fusion
97	37	Male	-0.2	L4/5 disc herniation	Disctomy and spinal fusion
98	35	Male	0.8	L5/S1 disc herniation	Disctomy and spinal fusion
99	38	Female	-1.4	Lumbar spinal stenosis and disc herniation (L4/5)	Laminectomy and spinal fusion
100	34	Male	0.6	L4/5 disc herniation	Disctomy and spinal fusion
101	35	Female	-0.7	L5/S1 disc herniation	Disctomy and spinal fusion

102	32	Male	1.0	L4/5 disc herniation	Disctomy and spinal fusion
103	39	Male	-1.5	Lumbar spinal stenosis and disc herniation (L5/S1)	Laminectomy and spinal fusion
104	34	Male	0.7	L4/5 disc herniation	Disctomy and spinal fusion
105	34	Female	0.2	L4/5 disc herniation	Disctomy and spinal fusion
106	36	Male	-0.3	L4/5 disc herniation	Disctomy and spinal fusion
107	38	Female	-1.3	Lumbar spinal stenosis and disc herniation (L4/5)	Laminectomy and spinal fusion
108	34	Male	0.5	L4/5 disc herniation	Disctomy and spinal fusion
109	35	Male	-0.4	L5/S1 disc herniation	Disctomy and spinal fusion
110	36	Female	-1.1	L4/5 disc herniation	Disctomy and spinal fusion
111	39	Male	-0.8	Lumbar spinal stenosis and disc herniation (L4/5)	Laminectomy and spinal fusion
112	31	Male	1.4	L4/5 disc herniation	Disctomy and spinal fusion
113	32	Female	-0.3	L4/5 disc herniation	Disctomy and spinal fusion

Supplementary Table 3. Top 100 small molecules selected from Apollo Scientific library

Zinc Code	Affinity	Lipinski Score	Lead Score	Rank	Molecule Information
ZINC03852644	-7.6	7	5	1	http://zinc.docking.org/substance/3852644
ZINC04277068	-7.4	7	5	2	http://zinc.docking.org/substance/4277068
ZINC19313632	-7.4	7	5	3	http://zinc.docking.org/substance/19313632
ZINC00154376	-7.4	7	5	4	http://zinc.docking.org/substance/154376
ZINC00089168	-7.4	7	5	5	http://zinc.docking.org/substance/89168
ZINC02574073	-7.3	7	5	6	http://zinc.docking.org/substance/2574073
ZINC02577549	-7.3	7	5	7	http://zinc.docking.org/substance/2577549
ZINC00096648	-7.3	7	5	8	http://zinc.docking.org/substance/96648
ZINC05278453	-7.2	7	5	9	http://zinc.docking.org/substance/5278453
ZINC00084031	-7.2	7	5	10	http://zinc.docking.org/substance/84031
ZINC06740830	-7.2	7	5	11	http://zinc.docking.org/substance/6740830
ZINC06732140	-7.2	7	5	12	http://zinc.docking.org/substance/6732140
ZINC00137445	-7.2	7	5	13	http://zinc.docking.org/substance/137445
ZINC02513020	-7.2	7	5	14	http://zinc.docking.org/substance/2513020
ZINC19850476	-7.2	7	5	15	http://zinc.docking.org/substance/19850476
ZINC02584341	-7.2	7	5	16	http://zinc.docking.org/substance/2584341
ZINC02574069	-7.2	7	5	17	http://zinc.docking.org/substance/2574069
ZINC08577839	-7.2	7	5	18	http://zinc.docking.org/substance/8577839
ZINC00160525	-7.1	7	5	19	http://zinc.docking.org/substance/160525
ZINC00162571	-7.1	7	5	20	http://zinc.docking.org/substance/162571
ZINC00150799	-7.1	7	5	21	http://zinc.docking.org/substance/150799
ZINC12368464	-7.1	7	5	22	http://zinc.docking.org/substance/12368464
ZINC05900737	-7.1	7	5	23	http://zinc.docking.org/substance/5900737
ZINC16159954	-7.1	7	5	24	http://zinc.docking.org/substance/16159954

ZINC08729782	-7.1	7	5	25	http://zinc.docking.org/substance/8729782
ZINC12336371	-7.1	7	5	26	http://zinc.docking.org/substance/12336371
ZINC20391245	-7.1	7	5	27	http://zinc.docking.org/substance/20391245
ZINC01529772	-7.1	7	5	28	http://zinc.docking.org/substance/1529772
ZINC02756301	-7.1	7	5	29	http://zinc.docking.org/substance/2756301
ZINC02243169	-7.1	7	5	30	http://zinc.docking.org/substance/2243169
ZINC02513039	-7.1	7	5	31	http://zinc.docking.org/substance/2513039
ZINC02574059	-7.1	7	5	32	http://zinc.docking.org/substance/2574059
ZINC03883871	-7.1	7	5	33	http://zinc.docking.org/substance/3883871
ZINC02568865	-7.1	7	5	34	http://zinc.docking.org/substance/2568865
ZINC02528960	-7.1	7	5	35	http://zinc.docking.org/substance/2528960
ZINC02574057	-7.1	7	5	36	http://zinc.docking.org/substance/2574057
ZINC00163794	-7	7	5	37	http://zinc.docking.org/substance/163794
ZINC08729986	-7	7	5	38	http://zinc.docking.org/substance/8729986
ZINC18100358	-7	7	5	39	http://zinc.docking.org/substance/18100358
ZINC00097218	-7	7	5	40	http://zinc.docking.org/substance/97218
ZINC00164277	-7	7	5	41	http://zinc.docking.org/substance/164277
ZINC02559799	-7	7	5	42	http://zinc.docking.org/substance/2559799
ZINC00111037	-7	7	5	43	http://zinc.docking.org/substance/111037
ZINC02572704	-7	7	5	44	http://zinc.docking.org/substance/2572704
ZINC15020910	-7	7	5	45	http://zinc.docking.org/substance/15020910
ZINC02565512	-7	7	5	46	http://zinc.docking.org/substance/2565512
ZINC02574072	-7	7	5	47	http://zinc.docking.org/substance/2574072
ZINC02574075	-7	7	5	48	http://zinc.docking.org/substance/2574075
ZINC02574862	-7	7	5	49	http://zinc.docking.org/substance/2574862
ZINC00168462	-7	7	5	50	http://zinc.docking.org/substance/168462

ZINC00168826	-7	7	5	51	http://zinc.docking.org/substance/168826
ZINC02522308	-7	7	5	52	http://zinc.docking.org/substance/2522308
ZINC33378823	-7	7	5	53	http://zinc.docking.org/substance/33378823
ZINC19850948	-7	7	5	54	http://zinc.docking.org/substance/19850948
ZINC33381070	-7	7	5	55	http://zinc.docking.org/substance/33381070
ZINC00035539	-7	7	5	56	http://zinc.docking.org/substance/35539
ZINC04419781	-7	7	5	57	http://zinc.docking.org/substance/4419781
ZINC00236402	-7	7	5	58	http://zinc.docking.org/substance/236402
ZINC02512536	-7	7	5	59	http://zinc.docking.org/substance/2512536
ZINC00317836	-7	7	5	60	http://zinc.docking.org/substance/317836
ZINC02509680	-7	7	5	61	http://zinc.docking.org/substance/2509680
ZINC04692806	-7	7	5	62	http://zinc.docking.org/substance/4692806
ZINC02382451	-7	7	5	63	http://zinc.docking.org/substance/2382451
ZINC06557742	-7	7	5	64	http://zinc.docking.org/substance/6557742
ZINC00155671	-7	7	5	65	http://zinc.docking.org/substance/155671
ZINC06732250	-7	7	5	66	http://zinc.docking.org/substance/6732250
ZINC00166760	-7	7	5	67	http://zinc.docking.org/substance/166760
ZINC00137063	-6.9	7	5	68	http://zinc.docking.org/substance/137063
ZINC00152628	-6.9	7	5	69	http://zinc.docking.org/substance/152628
ZINC36294169	-6.9	7	5	70	http://zinc.docking.org/substance/36294169
ZINC02561081	-6.9	7	5	71	http://zinc.docking.org/substance/2561081
ZINC15020915	-6.9	7	5	72	http://zinc.docking.org/substance/15020915
ZINC00157472	-6.9	7	5	73	http://zinc.docking.org/substance/157472
ZINC00129045	-6.9	7	5	74	http://zinc.docking.org/substance/129045
ZINC00158160	-6.9	7	5	75	http://zinc.docking.org/substance/158160
ZINC02525620	-6.9	7	5	76	http://zinc.docking.org/substance/2525620

ZINC02525598	-6.9	7	5	77	http://zinc.docking.org/substance/2525598
ZINC19851200	-6.9	7	5	78	http://zinc.docking.org/substance/19851200
ZINC02547487	-6.9	7	5	79	http://zinc.docking.org/substance/2547487
ZINC00162432	-6.9	7	5	80	http://zinc.docking.org/substance/162432
ZINC02574058	-6.9	7	5	81	http://zinc.docking.org/substance/2574058
ZINC02574060	-6.9	7	5	82	http://zinc.docking.org/substance/2574060
ZINC02574067	-6.9	7	5	83	http://zinc.docking.org/substance/2574067
ZINC02574071	-6.9	7	5	84	http://zinc.docking.org/substance/2574071
ZINC00317837	-6.9	7	5	85	http://zinc.docking.org/substance/317837
ZINC00404054	-6.9	7	5	86	http://zinc.docking.org/substance/404054
ZINC00086535	-6.9	7	5	87	http://zinc.docking.org/substance/86535
ZINC03883634	-6.9	7	5	88	http://zinc.docking.org/substance/3883634
ZINC01258204	-6.9	7	5	89	http://zinc.docking.org/substance/1258204
ZINC04222956	-6.9	7	5	90	http://zinc.docking.org/substance/4222956
ZINC00111588	-6.9	7	5	91	http://zinc.docking.org/substance/111588
ZINC06732251	-6.9	7	5	92	http://zinc.docking.org/substance/6732251
ZINC06732139	-6.9	7	5	93	http://zinc.docking.org/substance/6732139
ZINC12957994	-6.9	7	5	94	http://zinc.docking.org/substance/12957994
ZINC00153747	-6.8	7	5	95	http://zinc.docking.org/substance/153747
ZINC02525750	-6.8	7	5	96	http://zinc.docking.org/substance/2525750
ZINC05934541	-6.8	7	5	97	http://zinc.docking.org/substance/5934541
ZINC02525668	-6.8	7	5	98	http://zinc.docking.org/substance/2525668
ZINC04202596	-6.8	7	5	99	http://zinc.docking.org/substance/4202596
ZINC04023716	-6.8	7	5	100	http://zinc.docking.org/substance/4023716

Supplementary Table 4. Theoretical parameters of interaction and residues involved

Mode	Affinity (kcal/mol)	Dist from best mode		Residues
		rmsd l.b.	rmsd u.b.	
1	-7.2	0.000	0.000	G248, R289, Y297
2	-6.0	8.287	10.247	R295, Y297
3	-6.0	8.272	12.431	T291, R295, R289
4	-5.8	13.938	15.463	W262, R295, R269
5	-5.6	6.923	9.494	Y297, R289
6	-5.3	15.577	17.192	Q247

Supplementary Table 5. Biochemistry and hematology parameter assays for the chalcone derivative

Groups	ALT (U/l)	AST (U/l)	BUN (mmol/l)	WBC ($10^3/mm^3$)	HGB (g/dl)	RBC ($10^6/mm^3$)	HCT (%)	PLT ($10^3/mm^3$)
Vehicle	70.5 ± 6.9	89.5 ± 10.3	7.6 ± 0.9	5.6 ± 1.1	14.5 ± 1.4	9.7 ± 1.6	46.7 ± 6.1	671.3 ± 77.7
2 mg	73.7 ± 9.1	87.9 ± 11.7	7.9 ± 1.2	5.2 ± 0.8	15.4 ± 1.9	9.5 ± 1.3	50.3 ± 7.1	689.5 ± 79.9
4 mg	74.2 ± 9.2	95.8 ± 10.8	7.0 ± 1.5	6.2 ± 1.2	16.1 ± 1.7	10.9 ± 1.1	53.2 ± 6.2	725.1 ± 84.4

ALT: alanine transaminase; AST: aspartate transaminase; BUN: blood urea nitrogen; WBC: white blood cell; HGB: haemoglobin; RBC: red blood cell; HCT: hematocrit; PLT: platelets.

Supplementary Table 6. Biochemistry and hematology parameter assays for $(DSS)_6$-chalcone derivative

Groups	ALT (U/l)	AST (U/l)	BUN (mmol/l)	WBC ($10^3/mm^3$)	HGB (g/dl)	RBC ($10^6/mm^3$)	HCT (%)	PLT ($10^3/mm^3$)
Single dose								
Vehicle	68.7 ± 8.3	87.7 ± 11.9	7.3 ± 0.8	5.1 ± 0.7	13.8 ± 1.8	9.2 ± 1.3	44.1 ± 5.4	688.4 ± 74.3
2.5 μmol/kg	72.9 ± 6.8	84.4 ± 11.4	6.8 ± 0.9	4.7 ± 0.9	14.3 ± 1.9	8.8 ± 1.1	46.3 ± 6.4	694.6 ± 77.9
5.0 μmol/kg	69.7 ± 8.3	93.3 ± 12.4	7.5 ± 1.1	5.3 ± 0.8	13.5 ± 1.6	9.8 ± 1.3	47.7 ± 7.5	715.4 ± 80.3
10.0 μmol/kg	74.5 ± 8.7	90.3 ± 11.4	6.5 ± 0.8	5.0 ± 0.9	14.5 ± 1.9	9.5 ± 1.2	44.3 ± 6.5	710.5 ± 86.7
15.0 μmol/kg	67.7 ± 8.1	95.1 ± 10.3	7.1 ± 0.9	5.4 ± 1.1	13.9 ± 2.1	10.2 ± 1.1	47.5 ± 7.3	720.3 ± 77.9
20.0 μmol/kg	70.6 ± 6.8	92.1± 11.5	7.4 ± 1.0	4.9 ± 1.0	14.9 ± 2.5	9.4 ± 1.4	49.3 ± 6.6	729.3 ± 64.3
Multiple doses								
Vehicle	70.8 ± 8.4	86.5 ± 8.9	7.5 ± 0.8	5.5 ± 0.8	14.2 ± 1.8	10.8 ± 1.5	46.3 ± 5.7	714.3 ± 86.7
10.0 μmol/kg	74.2 ± 8.3	91.3 ± 9.9	8.0 ± 1.2	5.9 ± 1.1	15.4 ± 2.2	11.2 ± 2.1	52.7 ± 6.9	728.2 ± 79.8

ALT: alanine transaminase; AST: aspartate transaminase; BUN: blood urea nitrogen; WBC: white blood cell; HGB: haemoglobin; RBC: red blood cell; HCT: hematocrit; PLT: platelets.
